# Fabrication of a PVA-encapsulated MCC/S–VO_2_ composite via melt intercalation for efficient fixed-bed adsorption of methylene blue

**DOI:** 10.1038/s41598-025-22645-4

**Published:** 2025-10-29

**Authors:** Mona S. NourEldien, Hisham M. Aly

**Affiliations:** Department of Chemistry, Faculty of Science, Benha University, Benha, 13518 Egypt

**Keywords:** Melt intercalation, PVA composite, Fixed-bed adsorption, MB removal, Breakthrough curves, Chemistry, Energy science and technology, Environmental sciences, Materials science

## Abstract

In this study, a novel multifunctional composite comprising polyvinyl alcohol (PVA), microcrystalline cellulose (MCC), sulfur, and vanadium dioxide (VO_2_) was successfully synthesized through a solvent-free melt intercalation method. The process involved dispersing MCC/S–VO_2_ material within a PVA matrix in the molten state, enabling homogeneous mixing and effective interfacial integration, and subsequently calcining the mixture at 300 °C. Calcination preserved the monoclinic phase of VO_2_ and enhanced the composite’s porosity through thermal decomposition of PVA and MCC, resulting in uniformly distributed active phases within black, granular adsorbents. X-ray diffraction (XRD) confirmed the sustained integrity of the crystalline monoclinic VO_2_ phase. X-ray photoelectron spectroscopy (XPS) detected the elements carbon, oxygen, sulfur, and vanadium, along with carbonized polyvinyl alcohol (PVA), all exhibiting distinct spectral characteristics that point to strong interfacial interactions within the composite. Scanning and transmission electron microscopy (SEM and TEM) revealed a porous, layered architecture with VO_2_ nanosheets uniformly distributed throughout the carbon-rich matrix. Continuous fixed-bed column experiments were conducted under varying operational parameters, including bed heights, initial methylene blue (MB) concentrations, and flow rates. The composite demonstrated exceptional adsorption efficiency, achieving a peak capacity of 47.7 mg g^−1^ under optimal conditions (bed height: 0.5 cm, MB concentration: 20 mg L^−1^, flow rate: 1 mL min^−1^, column diameter: 1 cm). Breakthrough curve analysis confirmed the validity of the BDST model for performance prediction (R^2^ > 0.9), whereas the Thomas and Yoon–Nelson models exhibited lower correlation coefficients (R^2^ < 0.9), suggesting reduced applicability under the tested parameters. Notably, the adsorbent retained over 83.6% of its initial adsorption capacity after four regeneration cycles, underscoring its structural resilience and recyclability. The adsorption mechanism for MB onto the synthesized composite was attributed to π–π interactions, hydrogen bonding, and electron donation from the amine groups of MB to vanadium sites. This study, for the first time, demonstrates the use of solid PVA in a solvent-free melt intercalation process to fabricate polymer–inorganic hybrid adsorbents, offering a novel and eco-friendly strategy for advanced wastewater treatment.

**Supplementary Information:**

The online version contains supplementary material available at 10.1038/s41598-025-22645-4.

## Introduction

The release of synthetic dye-laden wastewater into natural water bodies presents serious environmental and health concerns due to the persistent stability and elevated toxicity of these dyes. Their accumulation in ecosystems poses significant risks to both human health and aquatic life^1,2^. Industrial effluents often contain a diverse range of dyes, including azo, reactive, acidic, alkaline, and neutral types, that exhibit strong resistance to biodegradation. This persistence amplifies the threat, as many of these dyes are known to be toxic, teratogenic, carcinogenic, and mutagenic. Addressing these challenges and implementing effective control measures demands substantial time and effort^3,4^. While reliance on conventional wastewater treatment technologies remains complex and resource-intensive.

To mitigate dye pollution, various remediation techniques have been explored, including coagulation-flocculation^5^, photocatalysis^6^, membrane filtration^7^, and adsorption^8–10^. Among these, adsorption is regarded as a highly efficient and cost-effective method that minimizes the formation of harmful byproducts while achieving substantial dye removal. Its key advantages include operational simplicity, low energy demand, environmental sustainability, and potential for recyclability^11,12^. Continuous adsorption systems offer benefits in wastewater treatment, thanks to their economic viability, process efficiency, and multitasking capacity^13–16^. Although batch processes are widely studied, they often fall short in practical, large-scale applications. In contrast, fixed-bed column systems have emerged as a promising solution for industrial effluent treatment, offering superior performance and scalability^17^.

A pivotal factor in adsorption efficacy is the selection of the adsorbent material. Ideal adsorbents should be inexpensive, biodegradable, easily recoverable, and reusable^18^. However, powdered adsorbents used in column reactors present several operational drawbacks, mainly low mechanical stability, which can cause pressure drops, flow blockages, and inconsistent distribution, ultimately reducing adsorption performance^19,20^. Granulation strategies, especially those involving binding agents, offer a compelling alternative by enhancing structural integrity and durability, by improving the mechanical properties^21,22^.

Melt intercalation requires statically or shear-annealing a polymer-organically modified material mixture over its softening point^23^. Since it uses no solvents and is compatible with existing processes, it is ecologically friendly and cost-effective. Melt intercalation into granules represents a promising approach for synthesizing adsorbent composites tailored for fixed-bed applications. Studies on various granule types—including zeolites^24^, hydroxyapatite^25^, and modified fly ash^26^— have demonstrated effective pollutant removal, with adsorption performance modulated by factors such as bed height, flow rate, and initial contaminant concentration. Integrating granulation with melt intercalation thus provides a robust framework for developing durable, reusable adsorbents with enhanced column performance.

Polyvinyl alcohol (PVA), an affordable and non-toxic synthetic polymer, has proven to be an effective binder for granules used in wastewater treatment due to its mechanical strength, chemical stability, and thermal resilience^27^. PVA not only reinforces the structural integrity of the adsorbent but also preserves the surface functionality and high activity of embedded materials during adsorption processes. In fixed-bed systems, PVA facilitates the formation of stable granules with superior adsorption performance^28–30^. Additionally, when subjected to thermal treatment, PVA can act as a coating agent, yielding a porous carbon layer that enhances adsorption capacity^31^.

Building on our previous findings^6^, this study introduces—for the first time—the synthesis of granular MCC/S–VO_2_ composite using PVA as a solid-phase binder through a solvent-free melt intercalation method. This innovation represents a significant advancement in structured adsorbent design for fixed-bed column applications. Comprehensive performance evaluations were conducted under various operational conditions, focusing on bed height, flow rate, and methylene blue (MB) concentration. Elution tests further assessed the adsorbent’s regenerative capabilities and potential for reuse. Breakthrough behavior was modeled using Thomas, Yoon–Nelson, and Adams–Bohart kinetic frameworks to interpret the experimental data. Additionally, an adsorption mechanism was proposed based on structural and surface characterization analyses.

## Experimental methods

### Materials

All the chemicals were of analytical grade and used without further purification. Vanadium pentoxide 99.6% (V_2_O_5_), methylene blue (MB) dye, elemental sulfur 99.9% (S), and Diethanolamide (DEA) nonionic surfactant 99% were purchased from Sigma-Aldrich Chemical Co.; Cotton fibers from pharmacy (Note: Cotton fibers, V_2_O_5_, S, and DEA were used in the synthesis of the MCC/S–VO_2_ material as described in literature^6^. Hydrochloric acid 32% (HCl). Polyvinyl alcohol (PVA) DP (1700–1800) was supplied by Alpha Chemika Co.

### Preparation of PVA/MCC–S–VO_2_ composite via melt intercalation (dry mixing)

The PVA-coated adsorbent granules were principally derived from an MCC/S–VO_2_ nanocomposite, synthesized following the methodology outlined in our earlier published research^6^. The PVA/MCC–S–VO_2_ composite was synthesized using a solvent-free melt intercalation approach, a technique previously described for producing polymer–inorganic nanocomposites in similar systems^32^. In this study, the method was creatively adapted to develop a PVA-based hybrid composite incorporating MCC/S–VO_2_.

**Fig. 1:**
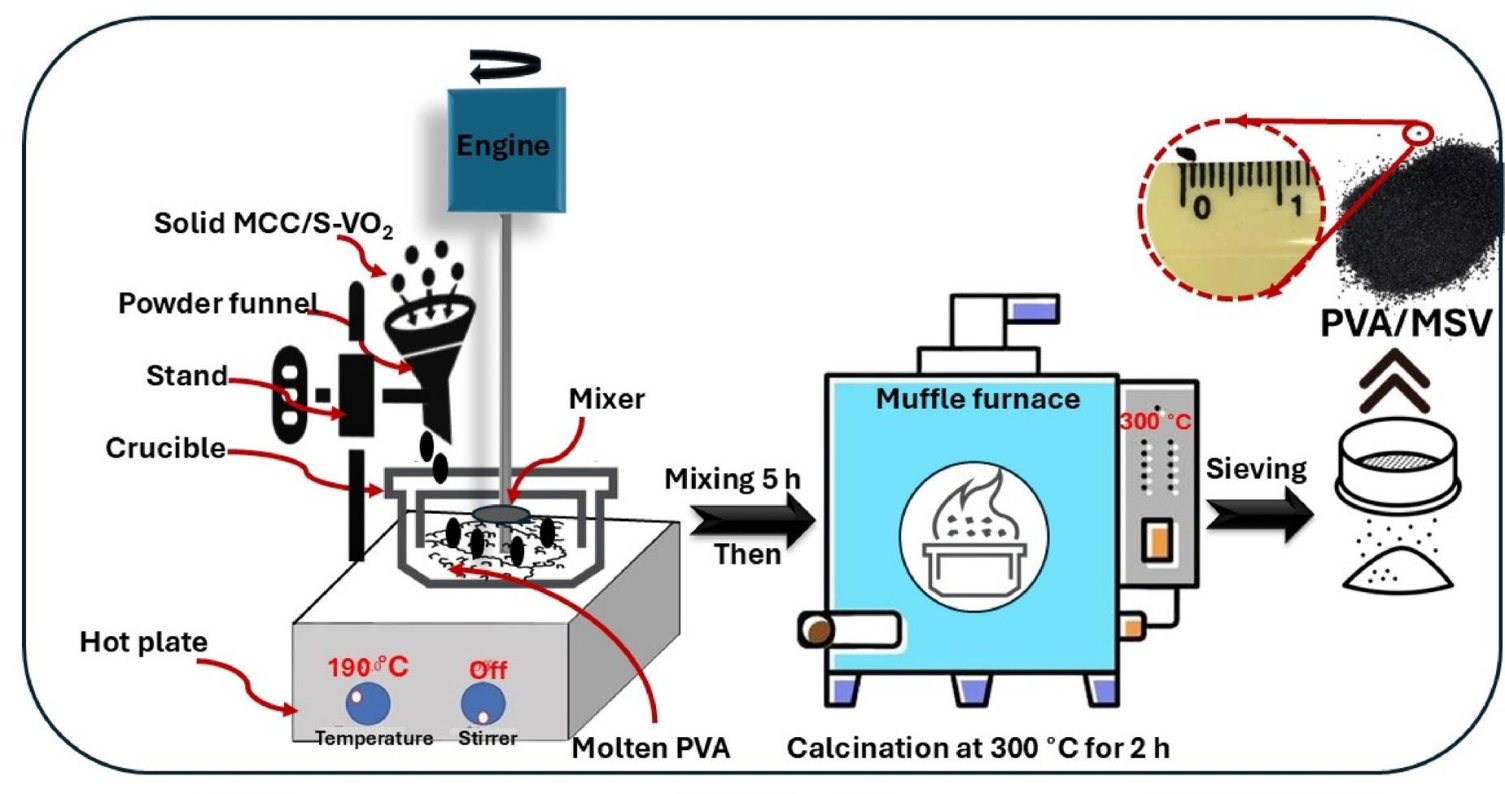
The comprehensive steps of preparation of PVA-MSV nanocomposite.

Solid PVA (10 g) was initially thermally softened by heating to 190 °C in a porcelain crucible under continuous stirring at 300 RPM for 10 min. Following this, a pre-blended MCC/S–VO_2_ mixture (5 g) was added at a fixed mass ratio of 2:1 (PVA: MCC/S–VO_2_). The mixture was maintained at 190 °C for 5 h to ensure homogeneous dispersion of the inorganic components within the polymer matrix. The used parameters (190 °C, 5 h, with stirring) were selected based on the thermal behavior of PVA, which softens at 190–200 °C without degradation^33^. These conditions ensured homogeneous dispersion of MCC/S–VO_2_, stable interfacial integration, and preservation of the crystalline VO_2_ phase. The resulting composite was collected, ground using a seasoned mortar, and subsequently calcined in air at 300 °C for 2 h to enhance porosity, promote partial carbonization of both PVA and MCC, and preserve the crystalline VO_2_ phase^34–36^. The final product was sieved to obtain granules measuring ~ 0.1 mm in diameter. The resulting composite is henceforth referred to as PVA/MSV, with MSV representing the MCC/S–VO_2_ component. Figure 1 outlines the detailed sequence of steps employed in the synthesis process.

### Instruments

The structure of the prepared samples was analyzed using a Bruker D8 Advance X-ray diffractometer (XRD) with Cu-Kα radiation and a step interval of 0.02° s^−1^. The full-width at half maximum (FWHM) values were corrected for instrumental broadening using a standard silicon (Si) sample. Functional groups were characterized through Fourier transform infrared (FT-IR) spectroscopy, using a Thermo Scientific Nicolet iS10 in the range of 4000–400 cm^−1^. Additionally, XPS measurements were carried out on a Thermo Fisher Scientific spectrometer equipped with a monochromatic Al Kα radiation source (hν = 1486.6 eV, resolution ~ 0.5 eV at pass energy 20 eV). Survey spectra were collected in the − 10 to 1350 eV rangespot size 400 μm, at a pressure of 10^−9^ mbar with full spectrum pass energy of 200 eV and at a narrow spectrum of 50 eV. A low-energy electron flood gun was used for charge neutralization during acquisition, and binding energies were calibrated with respect to the C 1 s peak at 284.8 eV. For peak fitting, spectra were processed using Gaussian–Lorentzian (GL) line shapes with Shirley background subtraction. The morphology was examined by field emission scanning electron microscopy (FE-SEM, Quanta 250 FEG) coupled with energy dispersive X-ray (EDX) at 30 kV, and high-resolution transmission electron microscopy (HR-TEM, JEM-2100) at 200 kV. N_2_ adsorption/desorption isotherms at 77 K were employed to examine the textural surface characteristics and pore size distribution using a BELSORP36 analyzer (JP. BEL Co., Ltd). Raman spectrometer (BRUKER-SENTERRA, Bruker Optics, Billerica, United States) equipped with an integral microscope (Olympos); the excitation source was Nd/YAl G laser (784 nm). The concentration of methylene blue (MB) dye was measured using a Jasco UV-visible spectrophotometer (V670) equipped with an integral sphere (ISN-723).

### Preparation of MB solution

A stock solution of 1000 mg L^−1^ was carefully prepared by dissolving 1 g of MB powder in one liter of distilled water. The specific requirements for the continuous setup were met by accurately achieving the desired concentrations of 20 mg L^−1^, 30 mg L^−1^, and 40 mg L^−1^ through a systematic dilution of the stock solution. This method was implemented to ensure reliability and uniformity in the experimental setup.

### Fixed-bed column adsorption studies

**Fig. 2:**
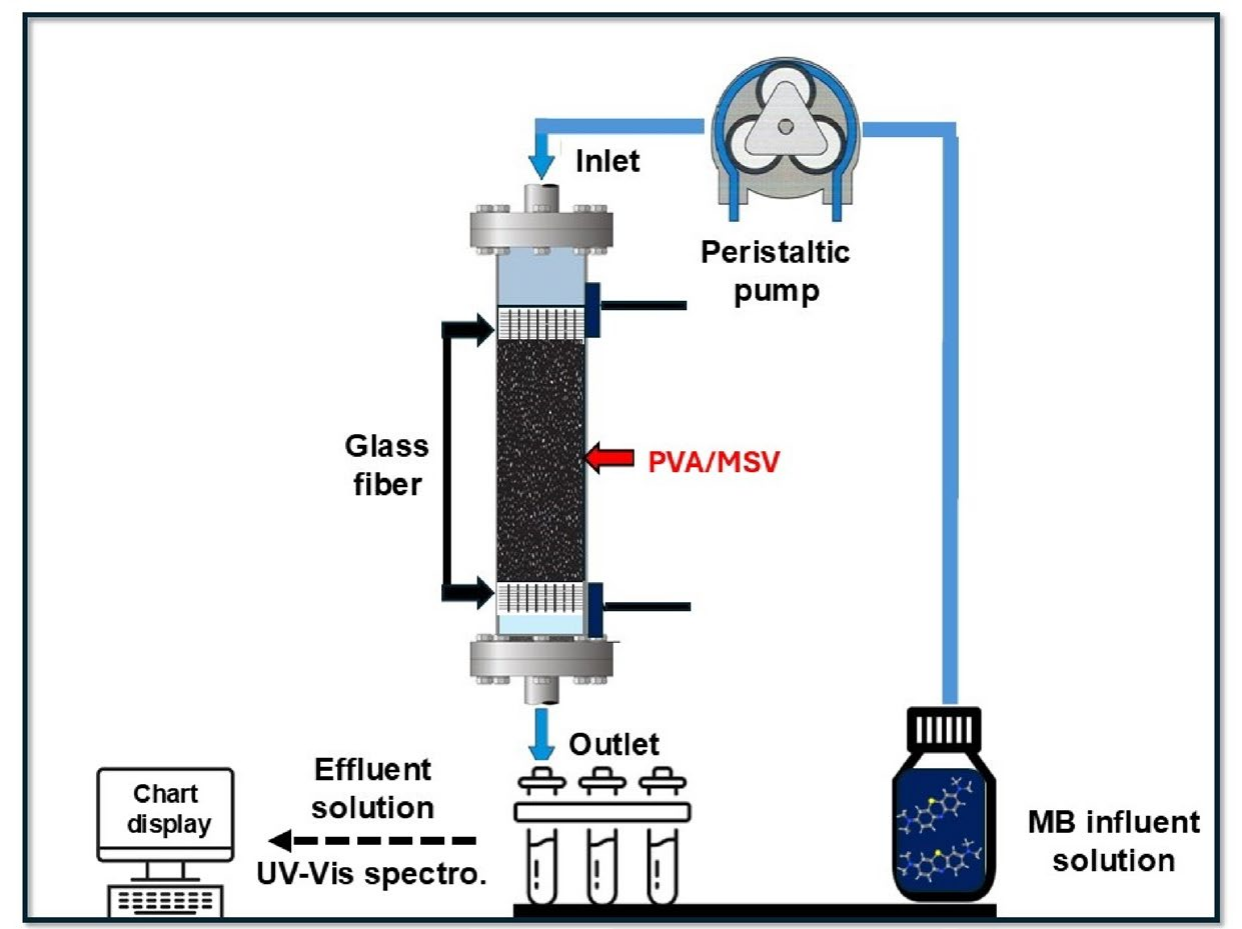
Fixed-bed column adsorption of MB dye onto PVA/MSV.

Adsorption experiments were conducted at ambient laboratory temperature (25 ± 2 °C) using a Perspex glass column with an inner diameter (i.d.) of 1 cm and 15 cm in length. The system maintained a pH range of 6.6–6.8, aligning with that of the methylene blue (MB) dye solution. Three key operational variables—initial dye concentration, flow rate, and bed depth—were systematically examined across separate column runs. Glass fiber supports were placed at both ends of the column to ensure consistent bed height throughout the tests. The column was packed with PVA/MSV composite (0.1 mm in granular size) in quantities of 0.1, 0.2, and 0.3 g, corresponding to bed depths of 0.5, 1, and 1.5 cm, respectively. MB solution with an inlet concentration of 20, 30, and 40 mg L^−1^ was introduced via a peristaltic pump at flow rates of 1, 1.5, and 2.5 mL min^−1^, as illustrated in Fig. 2. Effluent samples were collected periodically during preliminary experiments using a sample collector and analyzed spectrophotometrically at 664 nm. Adsorption continued until column saturation was achieved. Columns were wet-packed with the sorbent suspended in deionized water, and gentle vibration was applied to ensure uniform packing and minimize channeling or preferential flow paths^37^.

#### Selection of fixed-bed column operational parameters

Preliminary screening experiments were conducted to guide the choice of operational conditions for the fixed-bed column tests. Short breakthrough runs (~ 2 h duration) were carried out by varying one factor at a time while keeping the others constant. The ranges of bed height (0.5–1.5 cm), flow rate (1.0–2.5 mL min^−1^), and influent MB concentration (20–40 mg L^−1^) were selected to systematically probe the effects of residence time and adsorbent mass on breakthrough behavior while remaining within the linear detection range of UV–Vis. Screening results (Supplementary information (SI) Fig. S1) confirmed that increasing bed height delayed breakthrough, whereas increasing flow rate reduced contact time and advanced breakthrough. The corresponding empty bed contact times (EBCT) and breakthrough times (t_b_) are summarized in SI. Table S1. These observations provided the basis for selecting the detailed conditions employed in the main study.

### Fixed-bed column data analysis

The quantities and capacities of adsorption serve as critical metrics for success in column testing. Typically, researchers employ breakthrough curves to illustrate the performance of adsorbents within fixed-bed columns^38^. The abscissa of the breakthrough curve represents the running time (t) of the fixed bed, while the ordinate represents the ratio of effluent concentration (C_t_) to the influent concentration (C_0_) of the MB synthetic wastewater^38^. The adsorption capacity of the adsorbent under specific operational conditions can be determined from the breakthrough curve analysis. The breakthrough point is defined as the moment when the effluent concentration (C_t_) attains approximately 10% of the influent concentration (C_0_). The time associated is the breakthrough time (t_b_)^39^. When the effluent concentration reaches 90% of the influent concentration, this indicates the exhaustion point, and the corresponding duration is referred to as the exhaustion time (t_e_)^38,39^.

The total mass of adsorbates that have been adsorbed on PVA/MSV, denoted as q_total_ (mg), can be determined using Eq. (1)^40^.

1 $$\:{q}_{total}=\frac{F\:A}{1000}=\frac{F}{1000}{\int\:}_{t=0}^{t={t}_{total}}\left({c}_{0-}{c}_{t}\right)dt$$

where t_total_ is the total flow time (min), F is the flow rate (mL min^−1^), and A is the area above the breakthrough curve (cm^2^), (C_0_-C_t_) (mg L^−1^) is the adsorbed concentration.

The effluent volume, $\:\text{V}$_eff_ (mL), can be obtained using Eq. (2) as follows^41^:

2 $$\:{\:\:\:\:\:\:\:\:V}_{{e}_{ff}}=F\:{t}_{t}$$

where, $\:{\text{t}}_{\text{t}}$ (min) is the total flow time.

The equilibrium uptake q_eq_ (mg g^−1^), representing the maximum capacity of the column, is determined using Eq. (3) ^38^.

3 $$\:{q}_{eq}=\frac{{q}_{total}}{W}$$

where W is the dry weight of adsorbent in the column (g).

The empty bed contact time (EBCT) (min) was used to describe the average residence time of the influent in the packed bed and was calculated from Eq. (4)^42^:

4 $$\:EBCT=\frac{{V}_{b}}{Q}$$

where $\:{V}_{b}$​ is the bed volume (cm³), defined as the product of the column cross-sectional area and bed height, and $\:Q$ is the volumetric flow rate (mL min^−1^).

In each experiment, the mass transfer zone, MTZ (cm) was calculated by^43^

5 $$\:MTZ=L\left(1-\frac{{t}_{b}}{{t}_{sat.}}\right)$$

where *L* is the bed height (cm), t_b_ and t_sat_. are the breakthrough time and saturation time (min), respectively.

### Modeling of column data

Various adsorption models were utilized to predict the adsorption behavior of PVA/MSV over time. The models illustrate how the adsorbent material reacts under different conditions, providing critical insights for optimizing the fixed-bed column during the scaling-up process. The study employs three well-established mathematical models: the Adams–Bohart, Thomas, and Yoon–Nelson models.

A fundamental equation derived from surface reaction theory, as formulated by Bohart and Adams, illustrates the correlation between C_t_/C_0_ and t within a continuous system^44^. This model outlines the initial segment of the breakthrough curve and posits that equilibrium is not achieved instantaneously. This model assumes the negligibility of the resistances of external film and intra-particle diffusion type forces, so the adsorption kinetics are controlled by surface chemisorption between the adsorbate and the adsorbent with unused capacity^45^. These assumptions are reasonable in our system due to the small particle size, high porosity of the PVA/MCC–S–VO_2_ composite, and sufficiently high linear flow rate, which minimize diffusion limitations and ensure rapid equilibration at the particle surface. Hutchins developed the bed-depth service time (BDST) model, which correlated service time with fixed-bed height by linearizing the Bohart-Adams equation. The linear expression can be articulated as follows, Eq. (6)

6 $$\:t=\left(\frac{{N}_{0}}{{{C}_{0}U}_{0}}\right)Z-\frac{1}{{{C}_{0}k}_{{AB}}}{ln}\left(\frac{{C}_{0}}{{C}_{t}}-1\right)$$

Where C_0_ and C_t_ (mg L^−1^) represent the concentrations of dye at the input and outflow, respectively. The kinetic constant is represented as k_AB_ (L mg^−1^ min^−1^), whereas U_0_ (cm min^−1^) indicates the linear velocity, calculated by dividing the flow rate by the column section area (cm^2^). Z (cm) denotes the depth of the bed column, while N_0_ (mg L^−1^) represents the saturation concentration. The values of k_AB_ and N_0_ were derived from the intercept and slope of the linear plot of t against Z.

The Thomas model employs the Langmuir isotherm, integrating second-order reversible reaction kinetics and operating under the premise of plug flow behavior within the bed^46^. This well-established model elucidates the principles governing sorption processes in fixed-bed columns. This model is structured based on the adsorption processes, indicating that neither external nor internal diffusion serves as the limiting factor, as demonstrated in the subsequent linearized representation:

7 $$\:{ln}\left(\frac{{C}_{0}}{{C}_{t}}-1\right)=\frac{{k}_{TH}{q}_{e\:}m}{F}-{k}_{TH}{C}_{0}t$$

where $\:{\text{k}}_{\text{T}\text{H}}$ (mL mg^−1^ min^−1^) is the Thomas rate constant, $\:{\text{q}}_{\text{e}\:}$ (mg g^−1^) is the equilibrium loading capacity, $\:\text{F}\:$is the volumetric flow rate (mL min^−1^), and m (g) is the amount of adsorbent packed in the column. The values of $\:{\text{k}}_{\text{T}\text{H}}$ and $\:{\text{q}}_{\text{e}\:}$ can be determined from the slope and intercept of the linear plot of $\:\text{ln}\left(\frac{{\text{C}}_{0}}{{\text{C}}_{\text{t}}}-1\right)\:$against t.

The Yoon and Nelson model^47^ works under the assumption that the rate at which the likelihood of adsorbate molecule adsorption diminishes is directly related to both the probability of adsorbate adsorption and its breakthrough on the adsorbent^48^. In a system characterized by a single component, the linearized representation of the Yoon–Nelson model is articulated as follows:

8 $$\:{ln}\left(\frac{{C}_{t}}{{C}_{0}-{C}_{t}}\right)={k}_{YN}\left(t-\tau\:\right)$$

where, $\:{\text{k}}_{\text{Y}\text{N}}$ (min^−1^) is the Yoon-Nelson model constant; t (min) is the service time, and $\:{\uptau\:}$ (min) is the required time for 50% adsorbate breakthrough. The values of $\:{\text{k}}_{\text{Y}\text{N}}$ and $\:{\uptau\:}$ can be determined from the slope and intercept of the linear plot of $\:\text{ln}\left(\frac{{\text{C}}_{\text{t}}}{{\text{C}}_{0}-{\text{C}}_{\text{t}}}\right)\:$against t.

### Regeneration of the fixed bed column

Desorption experiments were performed by introducing a 0.1 M HCl solution as the eluting agent into the MB-saturated column. The solution was circulated for 30 min at a flow rate of 3 mL min^−1^. Afterward, the column underwent a 30-minute rinse with deionized water to prepare it for subsequent methylene blue adsorption. This adsorption–desorption cycle was repeated four times to evaluate the recyclability of the PVA/MSV composite. Regeneration efficiency was then calculated using Eq. (9):

9 $$\:Regeneration\:efficiency=\frac{{\left({q}_{e}\right)}_{r}}{{\left({q}_{e}\right)}_{0}}\times\:100$$

The reactivated column’s adsorption capacity is represented by (q_e_)_r_ (mg g^−1^), whereas (q_e_)_0_ indicates the initial capacity of the unused adsorbent (mg g^−1^).

**Ethical approval.** Institutional Review Board Statement: The study was conducted and approved according to the guidelines of the declaration of the ethical committee of the Faculty of Science, Benha University (no. BUFS-REC-2025-431Chm).

## Results and discussion

### Characterization of granular PVA/MSV

**Fig. 3:**
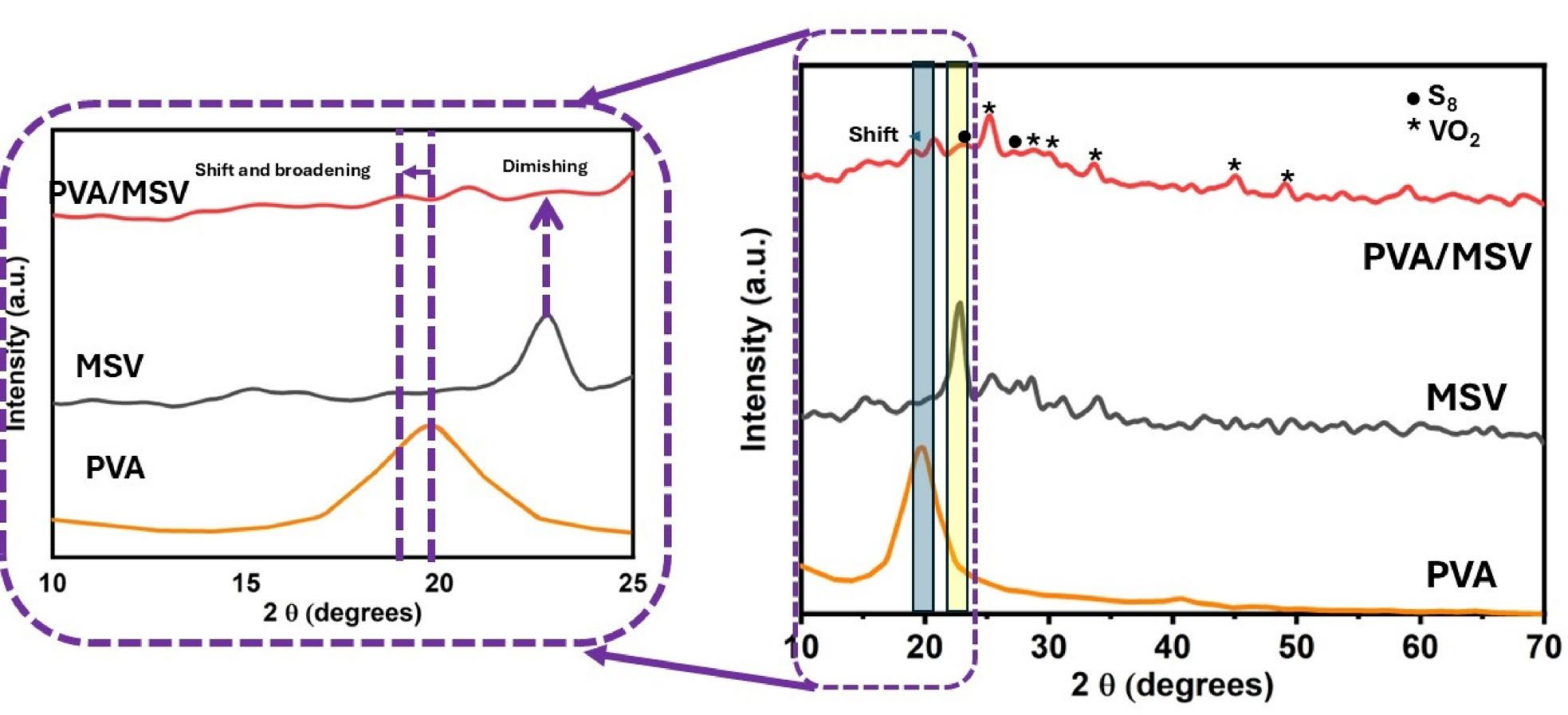
XRD patterns of PVA, MSV, and PVA/MSV nanocomposite.

X-ray diffraction analysis was employed to investigate the crystalline structures of pristine PVA, the MSV composite, and the melt-intercalated PVA/MSV hybrid, as depicted in Fig. 3. The diffraction profile of unmodified PVA revealed broad peaks centered at 19.5° and 38.6° (2θ), indicative of its semi-crystalline nature^49^. This property arises from the intermolecular hydrogen bonding and the orderly alignment of polymer chains within the PVA structure^50^. The MSV sample had several 15°–35° diffraction peaks. The indexed reflections show the monoclinic VO_2_ (M) phase, with the most prominent peaks marked with asterisks (*), confirming VO_2_’s crystalline integrity after sulfur dispersion and hydrothermal synthesis. Also, peaks related to elemental sulfur (S_8_) were discovered and highlighted with dots (●). Prior work has extensively explored the phase identification and peak assignment of the MSV composite, as has been done for polymeric nanocomposites^6^, and is presented here for comparative analysis. Upon incorporation of MSV into the PVA matrix via solvent-free melt intercalation, the diffraction pattern retained the characteristic peaks of both VO_2_ and S_8_ with a slight shift in the diffraction peaks, suggesting that the crystalline structure of the active components was maintained during the melt intercalation process. However, a noticeable broadening and shift of the PVA peak toward lower 2θ values was observed, which might be ascribed to the interaction between MSV and the PVA polymer blend. The results indicated a variation in the electrostatic interactions between PVA and the MSV, which modifies the blend and leads to an increase in the amorphous degree of the nanocomposite samples. Previous reports indicate analogous behavior for polymeric nanocomposites^51,52^. The yellow-highlighted region around 2θ ≈ 22.5° in the MSV pattern indicates a cellulose I structure, which is significantly diminished for the PVA/MSV sample. This change is attributed to the complete carbonization of MCC during the calcination process^35^. Thus, XRD data confirmed MSV dispersion in the PVA polymer blend matrix. Furthermore, the average crystallite sizes of PVA/MSV were calculated utilizing the Debye–Scherrer Eq. (10)^53^.

10 $$\:D=\frac{0.9\:\lambda\:}{\beta\:{cos}\theta\:}$$

where θ, , and are the Bragg diffraction angle, diffraction peak full width at half maximum (FWHM), and wavelength of the X-ray radiation (nm), respectively. The PVA/MSV composite has an average crystallite size of 21.35 ± 7.6 nm, lower than the virgin MSV material (34.76 ± 5.2 nm). The amorphous PVA phase and its strong interfacial interaction with MSV break PVA chain packing and cause structural distortion, reducing this. Literature describes similar behavior^54^.

**Fig. 4:**
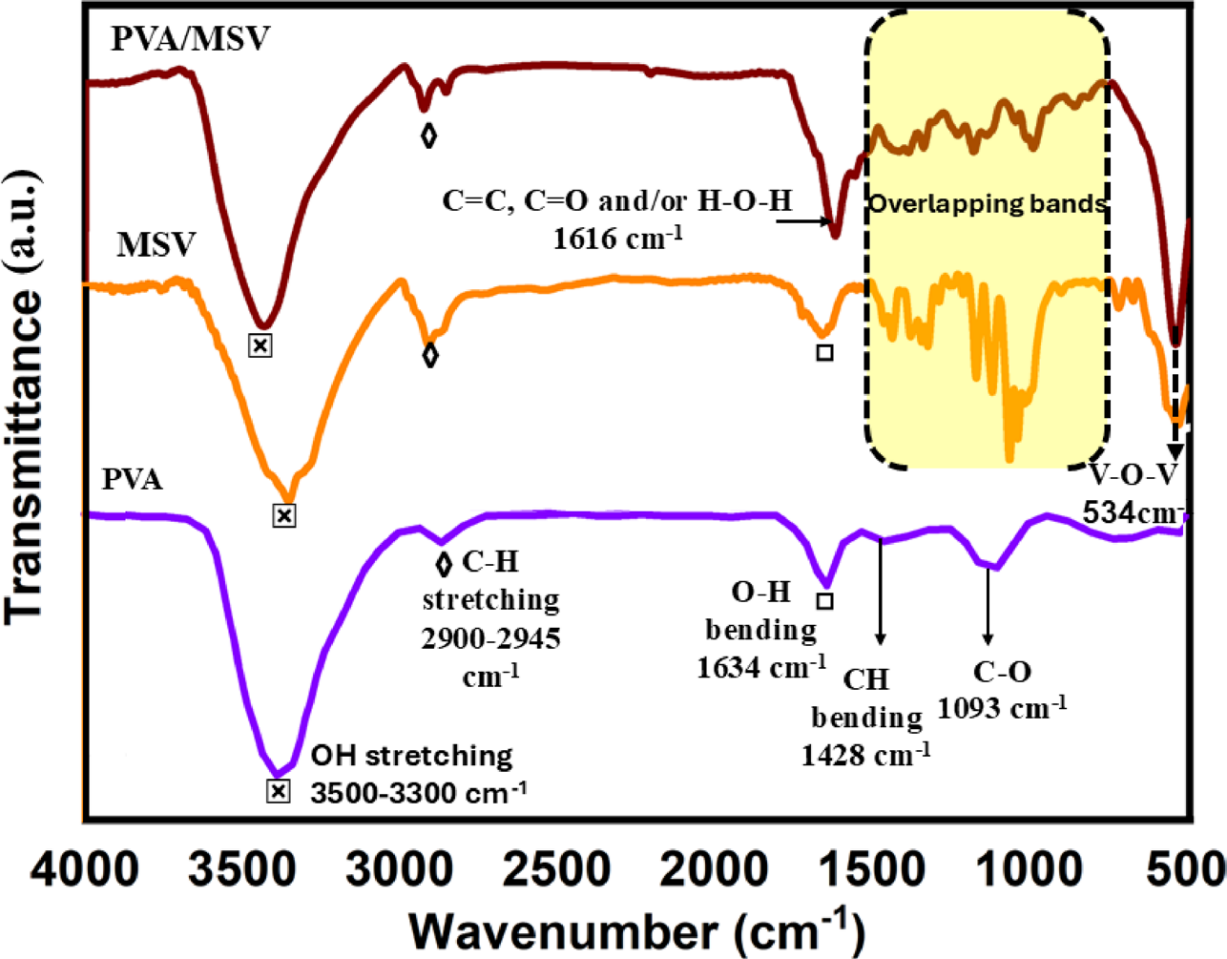
FTIR spectra of PVA, MSV, and PVA/MSV.

The FT-IR spectra of pure PVA, MSV, and the PVA/MSV composite are shown in Fig. 4. The results revealed substantial structural and chemical interactions during composite development. A broad absorption band from 3300 to 3500 cm^−1^ was observed in all spectra, indicating O–H stretching vibrations. The C–H stretching vibrations in the 2900–2945 cm^−1^ region were apparent in all spectra, primarily from PVA and/or MCC. A clear band at 1634 cm^−1^ implies O–H bending, while a new strong band at 1616 cm^−1^, commonly assigned to conjugated C = C stretching^55^, may also include contributions from H–O–H bending of adsorbed water carbonyl groups^56^ from partially hydrolyzed PVA. Porous and hydrophilic materials such as PVA, cellulose, or VO_2_ composites can readily re-adsorb atmospheric moisture, which may enhance this feature. Below 1600 cm^−1^, the yellow fingerprint region showed overlapping bands from C–O–C, C–H bending, and C–O stretching vibrations. The overlap makes peak assignment difficult, but it shows the components’ tight interaction in the organic matrix. The MSV and PVA/MSV samples showed a band at 534 cm^−1^, indicating the V–O–V bending vibrations in VO_2_, confirming its monoclinic inclusion^57^.

**Fig. 5:**
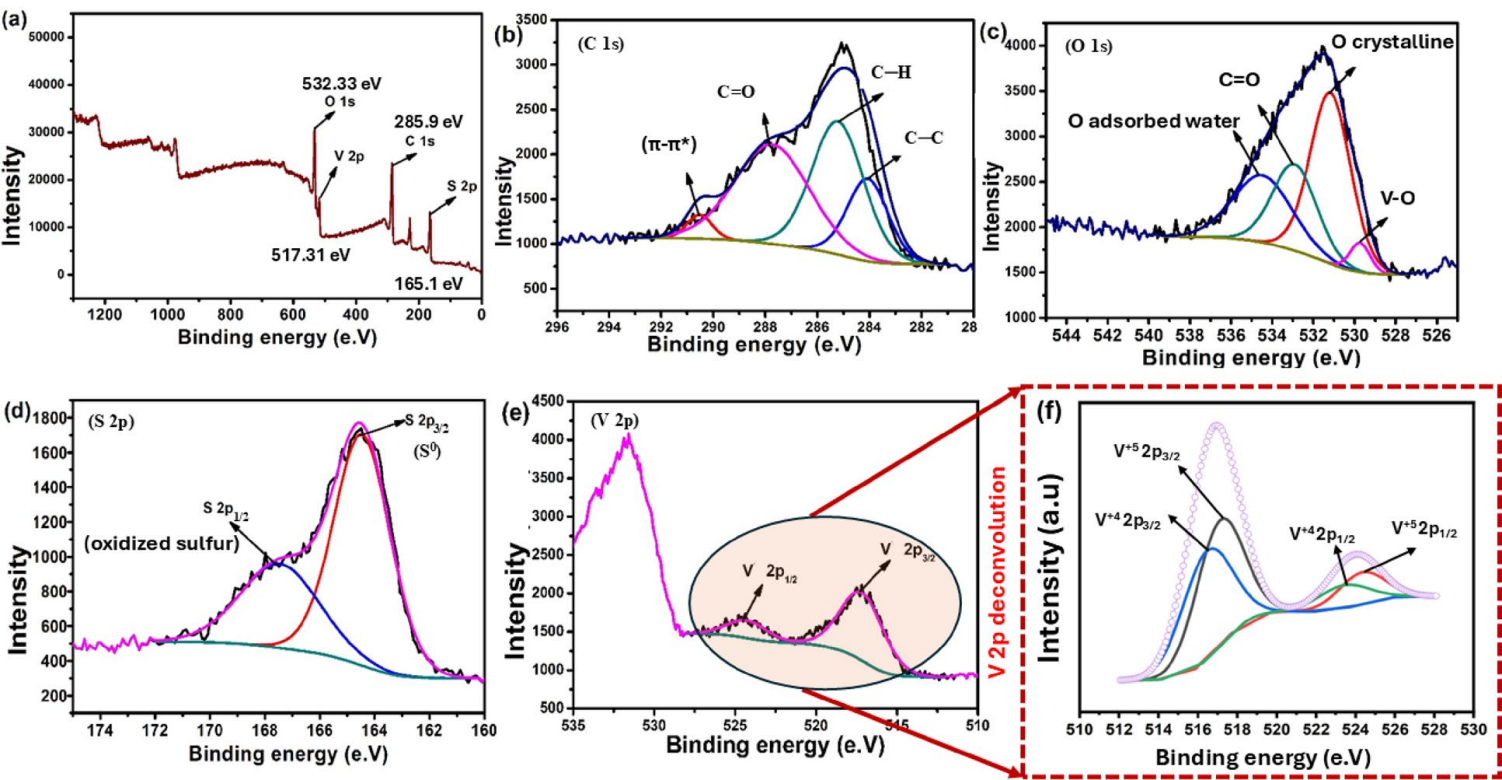
XPS results of PVA/MSV. XPS broad spectrum (a), XPS spectra of C 1 s (b), O 1 s (c), S 2p (d), V 2p (e), and the deconvoluted spectrum of V 2p (f) for PVA/MSV nanocomposite.

Figure 5a displays the XPS survey for the PVA/MSV, which indicates the presence of C, O, S, and V elements. C 1 s XPS spectrum (Fig. 5b) exhibited the superposition of four peaks, the first at 284.55 eV assigned to C─C bonds, the second one at 285.6 eV corresponded to C─H bonds^58^. The third peak at 287.87 eV was related to C = O^59^, and the fourth one at 291.2 eV was associated with π-π* transition of aliphatic C = C bonds of the thermally treated PVA^60^. However, the O 1 s broad peak could be deconvoluted to four peaks, Fig. 5c, corresponding to different oxygen atom environments. The peak at 529.73 eV corresponded to the lattice oxygen of VO_2_ (at the lowest BE), the peak at 531.18 eV might be attributed to O in the crystalline structure^61^. The peak at 532.4 eV corresponded to C = O, and the peak at 534.47 eV was related to the oxygen in the adsorbed water (at the highest BE). Figure 5d clearly showed two broadened peaks of S 2p at around 167.45, which were representative of oxidized sulfur species, such as sulfates or sulfonate^62^, and at 164.46 eV corresponding to elemental sulfur^63^. The V 2p spectrum (Fig. 5(e)) presents two main peaks at ~ 516.4 and ~ 523.5 eV, corresponding to V 2p_3/2_ and V 2p_1/2_, respectively. After deconvolution with Gaussian–Lorentzian functions and a Shirley background, the V 2p region was resolved into two doublets (Fig. 5(f)), each representing a different vanadium oxidation state. The fitting was performed with the 2p_1/2_–2p_3/2_ spin–orbit splitting constrained to a range of 7─7.4 eV. The dominant doublet at 517.11 eV (V 2p_3/2_) and 524.44 eV (V 2p_1/2_) is attributed to V^5+^, while a minor doublet at 516.43 eV (V 2p_3/2_) and 523.5 eV (V 2p_1/2_) corresponds to V^4+^. These assignments are consistent with literature values for VO_2_-type and V_2_O_5_-type vanadium oxides^64–67^. This analysis supported successful calcination and the retention of the core structural and chemical properties essential for adsorption. A summary of the fitting parameters, including binding energies, FWHM values, and relative areas, is provided in Table S2 (SI).

**Fig. 6:**
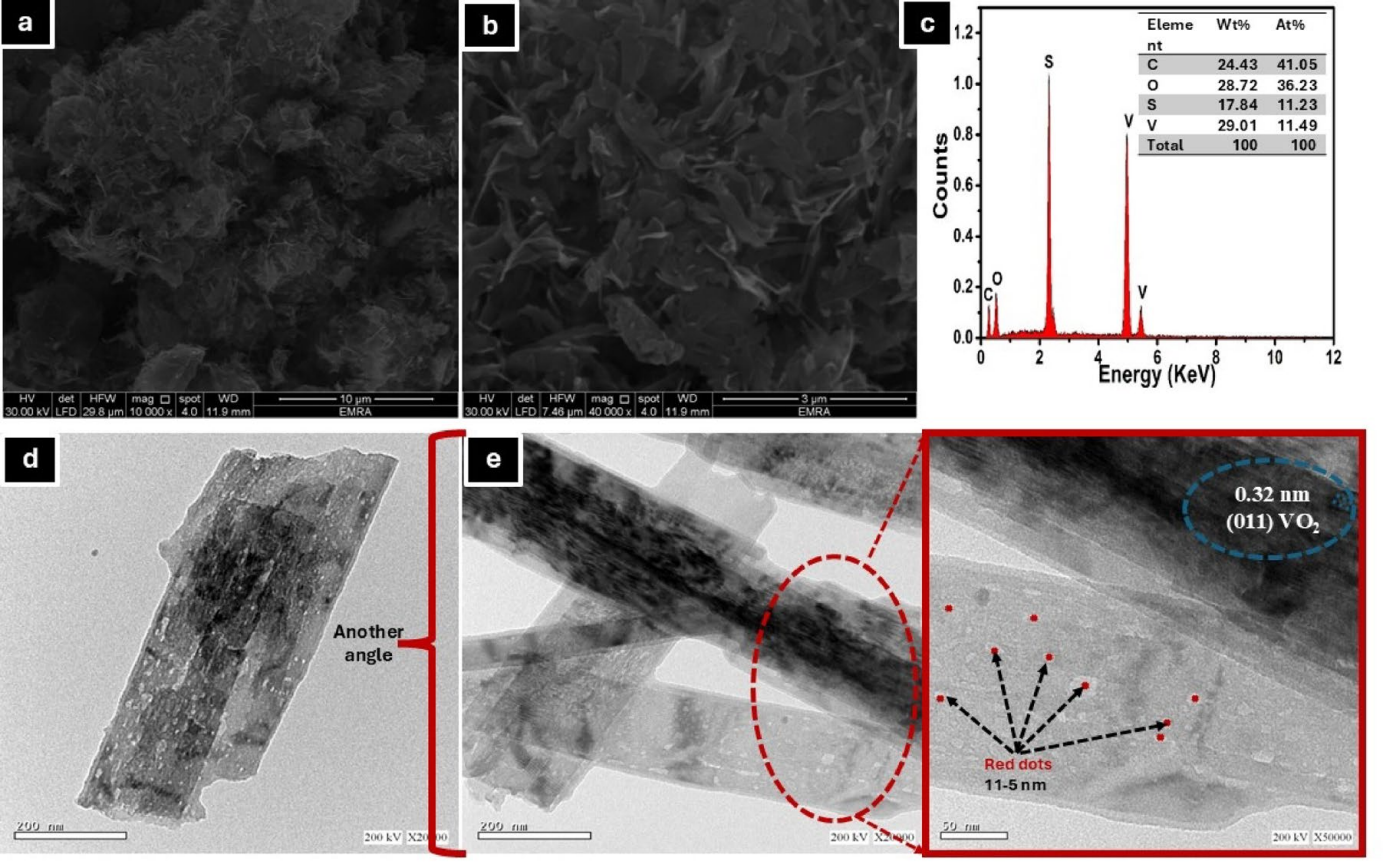
FE-SEM images at different magnifications (a-b), corresponding EDX data (c), and TEM (d-e) images of PVA/MSV.

FE-SEM was utilized to investigate the morphological alterations in the PVA matrix upon incorporation of the MSV composite. Figure 6 (a-b) presents the FE-SEM micrographs of the PVA/MSV composite. The surface of pure PVA appeared relatively smooth and uniform, indicative of its semi-crystalline nature and homogeneous film-forming ability^68^. In contrast, the PVA/MSV composite exhibited a significantly rougher and more heterogeneous surface morphology. The incorporation of MSV introduced notable textural changes, with the appearance of bright, dispersed domains corresponding to VO_2_-based nanoparticles with a thickness in the range of 20–40 nm embedded within the carbonized polymer matrix. These particles were uniformly distributed, reflecting good dispersion of the MSV phase throughout the PVA framework. This morphological change matched the self-contained MSV microstructure^6^ and verified hybridization. The rougher surface and linked porosity increase surface area and adsorption sites^69^, making them better for adsorption. EDX mapping can provide visual confirmation of the distribution of vanadium (V), oxygen (O), carbon (C), and sulfur (S) elements within the granules, as shown in Fig. 6c. Ideally, vanadium and oxygen should be evenly distributed, indicating good dispersion of the VO_2_ phase; possibly, sulfur from S-VO_2_ would be expected. In contrast, carbon would likely be more concentrated in the areas where PVA/MCC was originally present.

The TEM micrographs of the PVA/MSV composite (Fig. 6d-f) demonstrated the effective incorporation and distribution of VO_2_ nanostructures within the polymeric matrix. The VO_2_ particles primarily displayed nanosheet and rod-like shapes^6,70^, with a lateral size of 115–200 nm and a thickness range of 20–40 nm. These nanosheets contrast with the carbonaceous background, suggesting a consistent distribution within the PVA host. Nevertheless, the particles were not combined into a larger structure, despite being in contact with one another. A limited number of spherical or dot-like features were also present, probably indicating localized carbon residues that emerged during thermal processing. The distribution of these dots was sparse and did not suggest any notable clustering. This might be attributed to the thermal degradation behavior of PVA and MCC at 300 °C, where dehydration and depolymerization processes convert it into carbonaceous ash^34,35^. Constituent with SEM results, the uniform distribution of VO_2_ nanosheets within the PVA matrix prevents agglomeration, ensuring that a large fraction of the active surface sites remains accessible. This homogeneous dispersion shortens the diffusion path length for MB molecules, facilitates efficient surface complexation with vanadium centers, and thereby contributes to the high adsorption capacity observed in the fixed-bed column system.

The observed porous architecture, homogeneous dispersion of active S-VO_2_ nanosheets, and presence of carbonaceous residues indicated that the PVA/MSV composite possesses a high surface area and enhanced structural stability—key attributes that make it well-suited for adsorption-based applications, particularly in continuous fixed-bed systems.

**Fig. 7:**
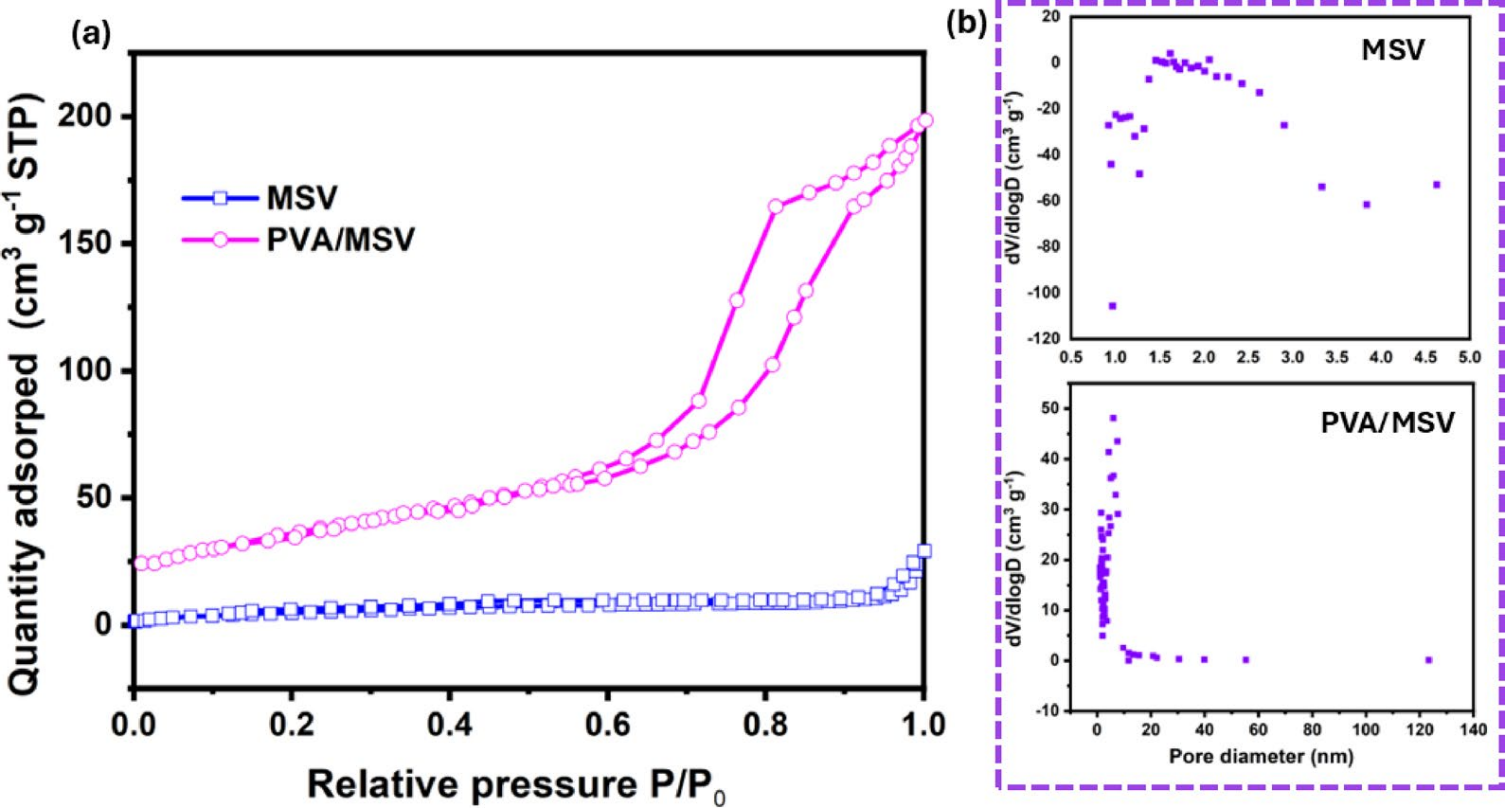
Nitrogen adsorption–desorption isotherms of MSV and PVA/MSV composites (a), and the corresponding pore size distribution curves (b).

To complement the morphological observations from SEM and TEM, BET surface area and pore size distribution analyses were performed (as shown in Fig. 7). The pristine MSV exhibited a surface area of 24.32 m^2^g^−1^ with an average pore diameter of mainly ~ 1.2–1.6 nm pores, confirming its microporous character. Upon incorporation into the PVA matrix, the PVA/MSV composite exhibited an increased surface area of 56.08 m^2^g^−1^, with dominant pores in the 3–5 nm range, confirming its mesoporous structure. This enhancement in surface area and porosity demonstrates that the composite structure promotes improved accessibility of active sites, thereby contributing to the higher adsorption capacity observed for MB.

### Fixed-bed column studies for the adsorption of MB over PVA/MSV nanocomposite

As described in the Methods, parameter ranges were first evaluated in short screening runs (SI. Fig. S1, Table S1), which confirmed the expected EBCT and breakthrough trends; these results guided the final choice of column conditions reported below. While these EBCTs are shorter than the 5–30 min range commonly reported for macro-scale dye adsorption studies, they were intentionally applied for micro-scale screening^71–73^. This approach is consistent with the Rapid Small-Scale Column Test (RSSCT) methodology, which uses short EBCTs to generate comparative adsorption data under accelerated conditions^74^. Nonetheless, for scale-up applications, longer EBCTs (≥ 10 min) should be evaluated to capture hydrodynamic and mass-transfer effects in larger columns.

Forecasting breakthrough curves with precision, considering specific operational factors, is crucial for the effective planning and management of a fixed-bed adsorption process. The performance of the PVA/MSV nanocomposite for the effective adsorption of MB in a fixed-bed column under different operational conditions was summarized in Table 1.

**Table 1:** Summary of operational column data parameters for the removal of methylene blue (MB) using the PVA/MSV nanocomposite.

Conc. (mg L^−1^)	Flow rate (mL min^−1^)	Bed height (cm)	Adsorbent mass (g)	t_b_ (min)	t_total_ (min)	V_eff_ (mL)	q_total_ (mg)	q_eq_ (mg g^−1^)	MTZ (cm)
20	1	0.5	0.1	61	591	591	4.77	47.7	0.45
20	1	1	0.2	104	767	767	7.18	35.9	0.86
20	1	1.5	0.3	182	1131	1131	12.75	42.5	1.26
20	1.5	1	0.2	27	477	715	5.29	26.5	0.94
20	2.5	1	0.2	17	300	750	5.06	25.3	0.94
30	2.5	1	0.2	6.8	167.5	418.5	3.08	15.4	0.96
40	2.5	1	0.2	4.75	122.5	306.3	1.83	9.15	0.96

#### Effect of initial MB concentration

**Fig. 8:**
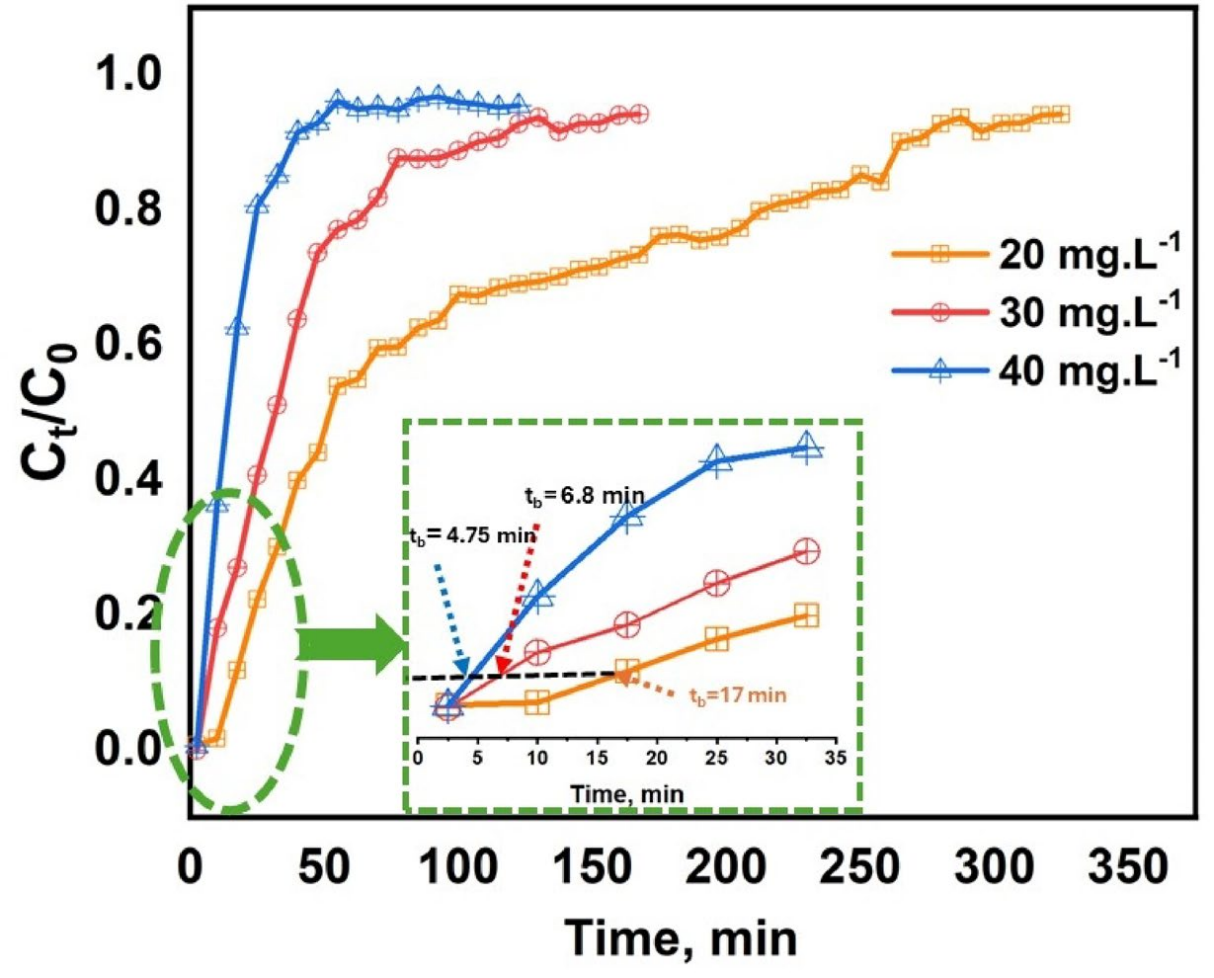
Breakthrough curves of MB dye in fixed-bed column packed onto PVA/MSV at different initial concentrations 20, 30, and 40 mg L^−1^: (bed depth = 1 cm, pH 6.6–6.8, flow rate = 2.5 mL min^−1^).

Figure 8 illustrates the complex interplay between the initial MB concentration and its resulting impact on the breakthrough curve. The research adjusted the initial concentration of MB between 20 and 40 mg L^−1^, while keeping the adsorbent column height at 1 cm, the feed flow rate at 2.5 mL min^−1^, and the pH level steady at 6.6–6.8. The figure illustrated that an increase in MB concentration accelerated the saturation of the adsorption process, leading to a reduction in breakthrough time from 17 to 4.75 min^75^. In contrast, at lower initial MB concentrations, one could observe a wider distribution of breakthrough curves and extended breakthrough times. Furthermore, with an increase in influent concentration, the loading rate of MB escalated, concurrently leading to a decrease in the length of the adsorption zone as a result of enhanced mass transfer driving forces^76^. In addition, higher concentrations slightly altered column hydrodynamics, where increased solution viscosity reduced the effective Reynolds number, resulting in sharper breakthrough profiles and faster bed saturation^77^.

#### Effect of PVA/MSV composite bed height

**Fig. 9:**
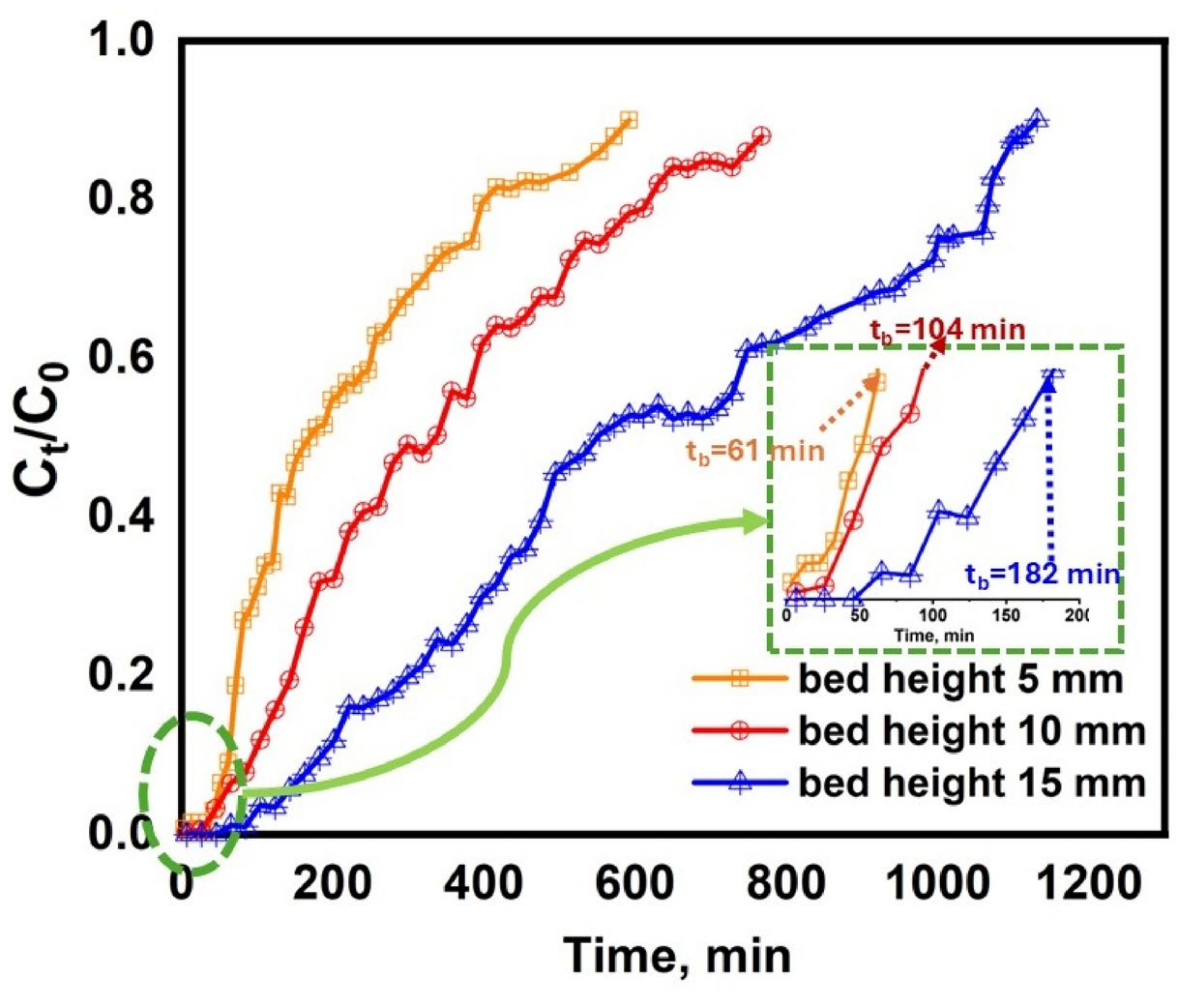
Breakthrough curves of MB dye in fixed-bed column packed onto PVA/MSV at different bed heights 5, 10, and 15 mm: (dye conc. = 20 mg L^−1^, pH 6.6–6.8, flow rate = 1 mL min^−1^).

The impact of PVA/MSV bed height on the breakthrough curve for MB adsorption was examined by varying bed heights of 0.5, 1, and 1.5 cm, while keeping the flow rate constant at 1 mL min^−1^, the initial MB concentration at 20 mg L^−1^, and the pH at 6.6–6.8. Figure 9; Table 1 demonstrated that an increase in column height resulted in a longer saturation period and a greater effluent volume, V_eff_. This phenomenon could be ascribed to a greater contaminant quantity needed to saturate a larger PVA/MSV bed, hence promoting an extended interaction between MB and PVA/MSV and improving removal efficiency^48^. The elevation of bed height resulted in a reduced slope of the breakthrough curve, signifying an expanded mass transfer zone^78^, hence leading to a more effective decrease in solute concentration due to the increased availability of adsorption sites^79^. To further quantify the bed height effect, the mass transfer zone (MTZ) was calculated based on breakthrough (t₀) and exhaustion times (tₑ) as shown in Table 1. The MTZ values were found to be 0.45 cm, 0.86 cm, and 1.26 cm for bed heights of 0.5, 1.0, and 1.5 cm, respectively. Although the MTZ length increased with bed depth, the relative ratio of MTZ to total bed height decreased from 0.90 to 0.86, and then to 0.84, suggesting sharper breakthrough fronts and more efficient utilization of the adsorbent bed at higher column depths. This trend indicates improved mass transfer performance when the bed height is increased.

#### Effect of the solution MB flow rate

**Fig. 10:**
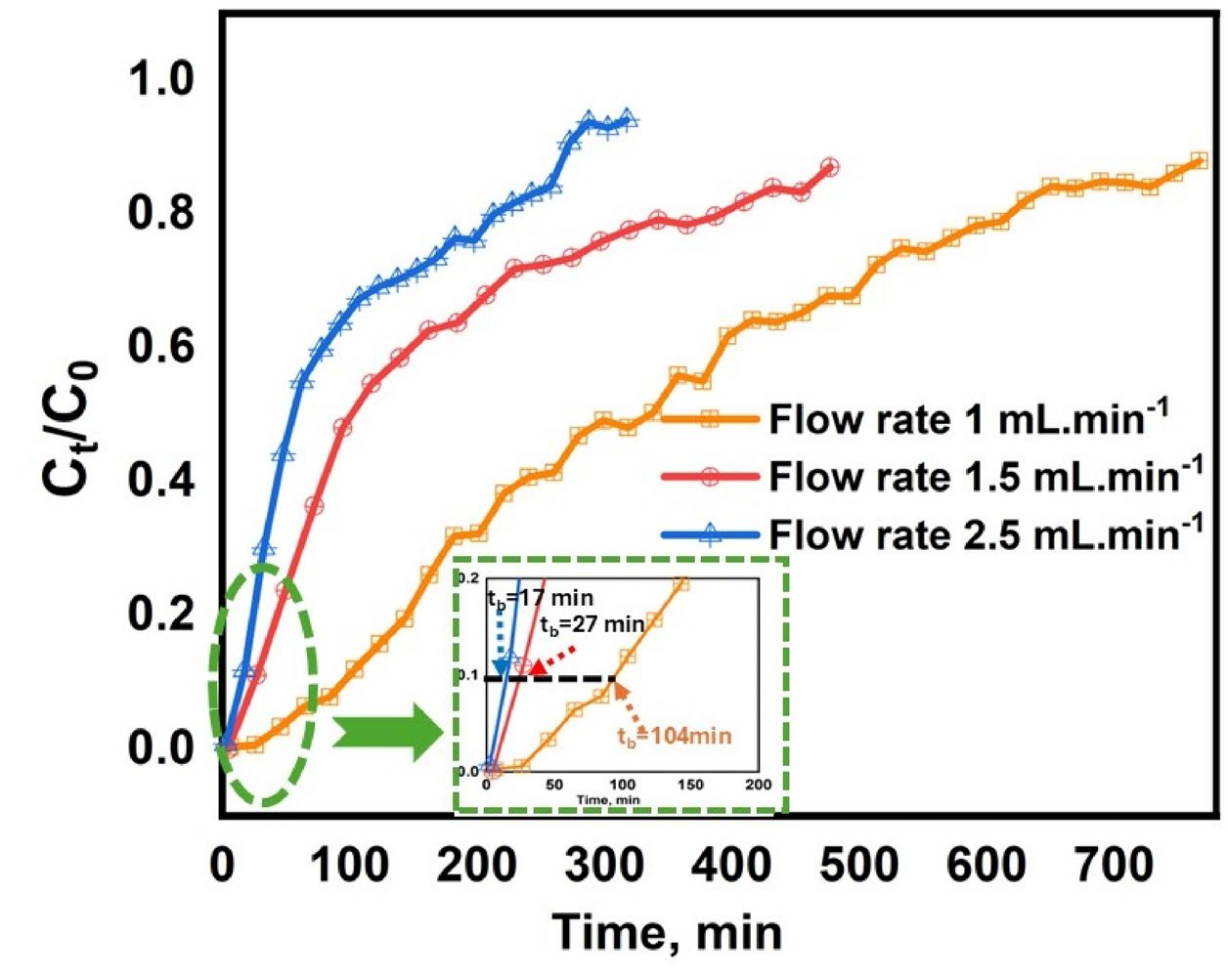
Breakthrough curves of MB dye in a fixed-bed column packed with PVA/MSV at different dye flow rates 1, 1.5, and 2.5 mL min^−1^: (dye conc. = 20 mg L^−1^, pH 6.6–6.8, bed height = 1 cm).

To assess the impact of flow rate on the breakthrough curve, the flow rate was adjusted from 1 to 2.5 mL min^−1^, while keeping the bed height (1 cm), initial MB concentration (20 mg L^−1^), and pH level (6.6–6.8) constant. Figure 10; Table 1 illustrate that increased flow rates led to reduced breakthrough times from 104 to 17 min. Conversely, reduced flow rates facilitated prolonged interaction between MB and PVA/MSV, leading to improved MB ion elimination and extended breakthrough times. The disparities in breakthrough curves could be ascribed to mass transfer principles, wherein elevated flow rates enhanced mass transfer, resulting in expedited saturation^78^. An elevated flow rate diminished removal effectiveness, since it failed to afford adequate residence time for the solute to engage with the sorbent within the column^80^. These observations align with findings reported by other researchers^48,81,82^. The potential influence of flow rate on channeling or preferential flow paths was carefully considered. The columns were packed using a wet-packing method with gentle vibration to ensure uniform bed density and minimize void formation. Moreover, the reproducibility and smooth profiles of the breakthrough curves at different flow rates indicate that the adsorption process was not significantly affected by channeling^37,83^.

#### Modeling

##### Bohart–Adams and bed-depth service time model (BDST)

**Fig. 11:**
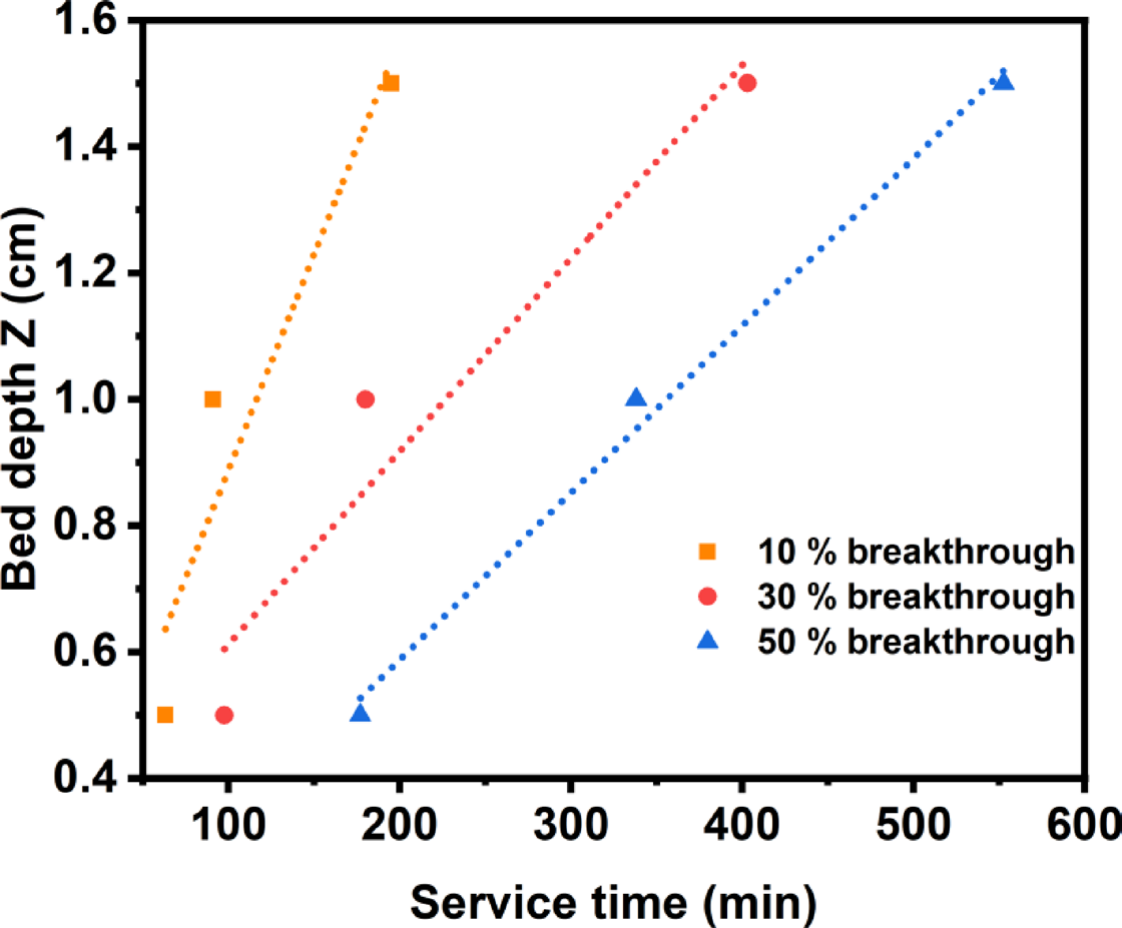
Linear regression plots of the BDST model at 10, 30, and 50% breakthrough for different bed heights.

To elucidate the adsorption kinetics and dynamic behavior of MB uptake on the PVA/MSV composite, the BDST (Bed-Depth Service Time) model was employed at several breakthrough points (10, 30, and 50%). The model parameters, specifically the adsorption rate constant (k_a_) and the maximum adsorption capacity (N_0_), were obtained and are presented in Table 2; Fig. 11 shows that the breakthrough curves shifted gradually with service time, demonstrating increasing adsorbent bed saturation. The adsorption capacity N_0_​ increased dramatically with greater breakthrough percentages, from 4914.54 mg L^−1^ (10%) to 13,591.04 mg L^−1^ (50%), showing improved column usage over time. The rate k_a_ fluctuated with the breakthrough level, indicating changing interaction dynamics in the adsorption zone. As breakthrough increases, the coefficient of determination (R^2^) improved to 0.986 at 50%, indicating a significant connection between experimental results and BDST predictions. These findings imply that the PVA/MSV composite has great potential for continuous-flow adsorption systems and that the BDST model is well-suited to describe its column performance.

**Table 2:** BDST model parameters at different bed heights.

Breakthrough (%)	10	30	50
k _a_ (L mg^−1^ min^−1^)	0.007561	0.001483	0.018876
N _0_ (mg L ^−1^ )	4914.543	11289.21	13591.04
R ^2^	0.9	0.934	0.986

##### Thomas model

**Fig. 12:**
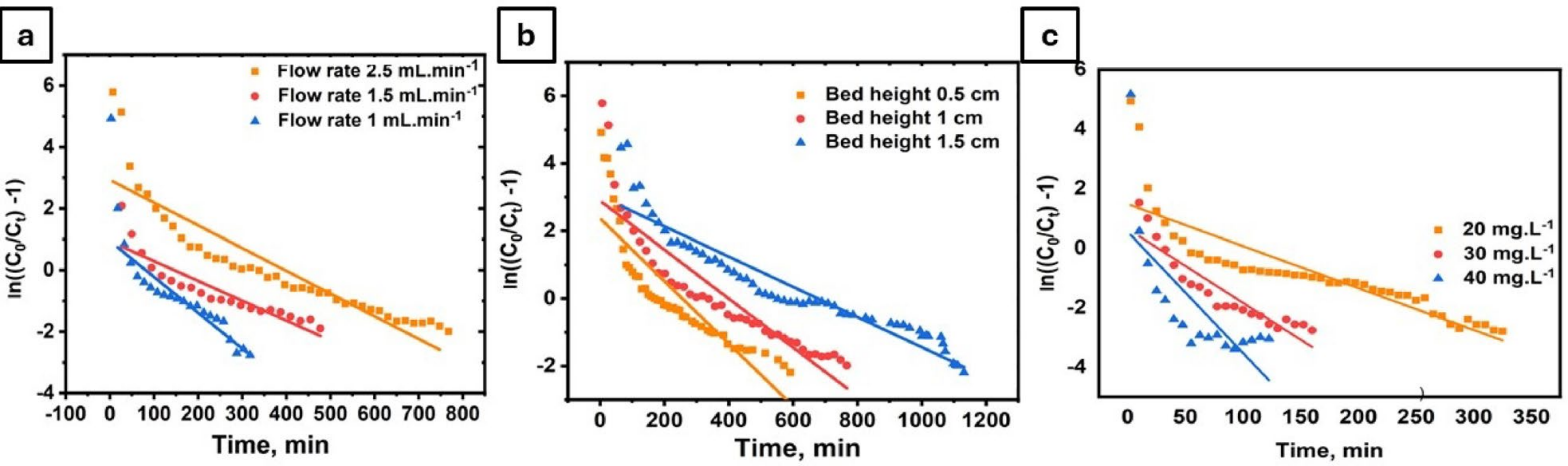
Thomas model-predicted breakthrough curves for MB adsorption onto PVA/MSV composite granules under varying flow rates (a), bed heights (b), and influent concentrations (c), under the same conditions as those used in the experimental runs.

The Thomas model is often used to predict breakthrough behavior in fixed-bed adsorption systems; however, this work appears to limit its usefulness. Table 3; Fig. 12 demonstrated that the model lacked a high correlation between experimental data and theoretical predictions, with all R^2^ values below 0.9. indicating that the model cannot completely describe MB adsorption on the PVA/MCC–S–VO_2_ composite. Although Thomas’ model assumes ideal plug flow with negligible axial dispersion and no significant intra-particle diffusion^84^. Deviations are likely due to axial dispersion along the column and intra-particle diffusion within the porous composite, which affect mass transfer and breakthrough behavior. Higher flow rates reduce contact time, leading to earlier saturation, while lower MB concentrations decrease the adsorption driving force^40^.

**Table 3:** Thomas model parameters for MB adsorption onto PVA/MSV at varying bed heights, flow rates, and influent concentrations.

Conc. (mg L^−1^)	Flow rate (mL min^−1^)	Bed height (cm)	Thomas parameters			
			K_th_ mL min^−1^ mg^−1^ × 10^−3^	q_Th_ mg g^−1^	q_eq_ cal. mg g^−1^	*R* ^2^
20	1	0.5	0.463	47.19	47.7	0.75594
20	1	1	0.362	38.16	35.9	0.81848
20	1	1.5	0.2235	27.96	42.5	0.89222
20	2.5	1	0.7035	26.18	25.3	0.73575
30	2.5	1	0.8266	9.74	15.4	0.84852
40	2.5	1	1.018	6.72	9.15	0.51818
20	1.5	1	0.322	21.86	26.5	0.82451

##### Yoon–Nelson model

**Fig. 13:**
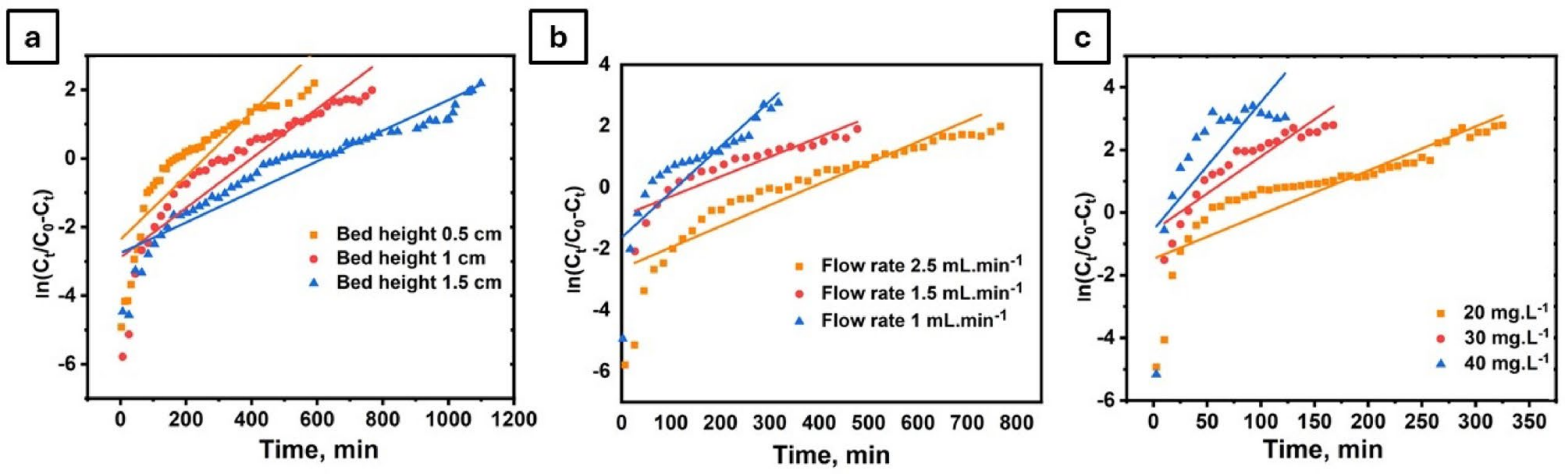
Yoon–Nelson model-predicted breakthrough curves for MB adsorption onto PVA/MSV composite granules under varying bed heights (a), flow rates (b), and influent concentrations (c), under the same conditions as those used in the experimental runs.

PVA/MSV granule breakthrough performance in methylene blue (MB) adsorption was assessed using the Yoon–Nelson model. According to Table 4; Fig. 13, the model showed moderate correlation coefficients (R^2^) from 0.51 to 0.89. The model failed to effectively predict breakthrough behavior in all experimental settings, especially when R^2^ < 0.9, even below 50% saturation. Due to the intricacy of the composite system’s sorption mechanisms and interactions, the Yoon–Nelson model may not be adequate for this investigation.

Overall, the marginal performance of both the Thomas and Yoon–Nelson models demonstrates that the adsorption of MB onto the PVA/MSV composite cannot be fully captured by single-step kinetic assumptions. Instead, the results indicate a more complex mechanism in which axial dispersion, intraparticle diffusion, and chemisorption interactions all contribute to breakthrough behavior.

**Table 4:** Yoon–Nelson model parameters for MB adsorption onto PVA/MSV at varying bed heights, flow rates, and influent concentrations.

Conc. (mg L^−1^)	Flow rate (mL min^−1^)	Bed height (cm)	Yoon-Nelson parameters			
			K_YN_ (min^−1^)	t_0.5_ YN (min)	t_0.5_ cal. (min)	*R* ^2^
20	1	0.5	0.00926	255.65	178	0.75594
20	1	1	0.00724	421	338	0.81848
20	1	1.5	0.00447	199.94	552.5	0.89352
20	2.5	1	0.01407	104.7	55	0.73575
30	2.5	1	0.02366	24.35	32.5	0.84319
40	2.5	1	0.04072	13.45	14	0.51818
20	1.5	1	0.00644	145.7764	113	0.82451

#### Desorption of MB

**Fig. 14:**
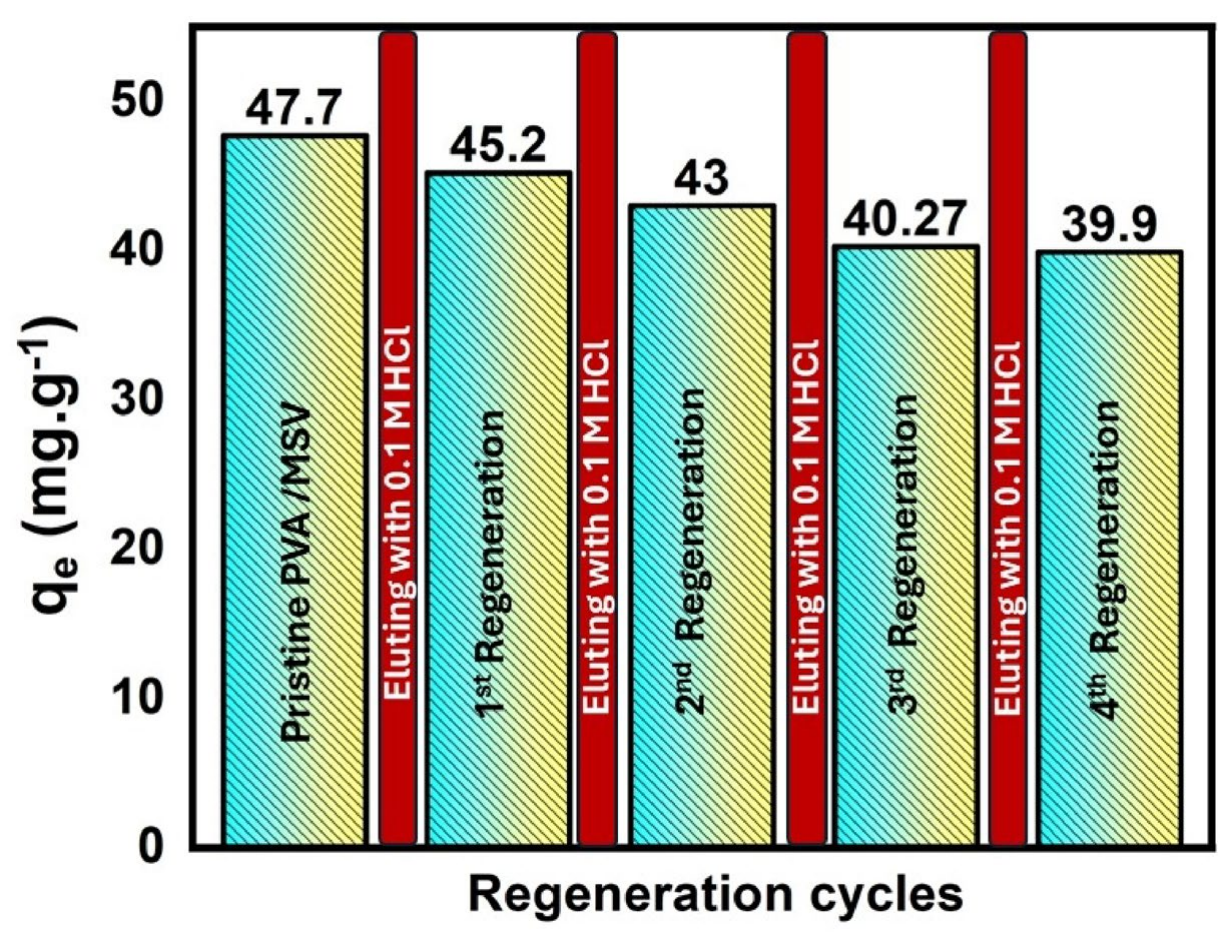
Regeneration experiments of PVA/MSV for the effective removal of methylene blue (MB): (dye conc. = 20 mg L^−1^, flow rate = 1 mL min^−1^, bed height = 0.5 cm).

Desorption studies were performed on the PVA/MSV sample saturated with MB to illustrate the recyclability of the PVA/MSV. The choice of HCl as regenerant was guided by preliminary screening experiments (SI. Fig. S2). In these tests, four eluents (0.1 M HCl, 0.1 M acetic acid, ethanol, and 0.1 M NaOH) were compared after one adsorption–desorption cycle. Acetic acid and HCl gave the highest percentages, whereas NaOH and ethanol were less effective. When reused in a fresh adsorption cycle, the HCl-regenerated adsorbent maintained the highest removal efficiency, confirming its superior performance. Therefore, HCl was selected as the regenerant for the detailed reusability experiments presented in the main study. The desorption method involved utilizing a 0.1 M HCl solution as the eluting agent. In an acidic environment, the active sites of the adsorbent material acquired protons, resulting in a reduction of the electrostatic interaction between the MB molecules and the active centers of the PVA/MSV. This decrease in attraction allowed MB molecules to migrate from the adsorbent material, facilitating their diffusion^85^. Figure 14 distinctly demonstrated that the adsorption efficiency of the PVA/MSV for MB progressively degrades after several reutilizations. After four cycles, the removal efficiency declined from 91.7% to 83.6%, which is mainly attributed to incomplete desorption of methylene blue (MB), partial pore blockage, and gradual loss of available surface sites during repeated reuse^86,87^. To address the observed decline in recyclability, additional regeneration protocols (SI. Fig. S3) were tested beyond the initial 0.1 M HCl circulation. Variants included (i) extended static soaking (50 min) and (ii) reduced-flow circulation (1.0 mL min^−1^) combined with 10 min ultrasonic assist. The optimized procedure (0.1 M HCl with 10 min sonication, DI rinse) improved regeneration efficiency across four cycles. These results indicate that extending contact time and introducing mild sonication enhance reusability, with recovery not reaching 100% which might be attributed to π–π aromatic interactions between methylene blue and the aromatic domains of the PVA/MSV. This interpretation is supported by the persistence of residual MB bands in FTIR (SI. Fig. S4), also the disappearance of π–π* peak in C 1 s spectrum and formation of new peaks related to N 1 s and Cl 2p (SI. Fig. S5). While SEM analysis (SI. Fig. S6) showed only minor changes in surface morphology even after optimized elution, consistent with incomplete desorption and gradual loss of accessible sites.

While a wide range of polymer-based systems have been explored, the use of PVA-based nanocomposites in fixed-bed column configurations remains notably limited. Concurrently, alternative adsorbent materials demonstrated comparable absorption capabilities. The incorporation of MCC/S-VO_2_ within the PVA synthesized via the melt intercalation method, then thermal treatment, improves the composite’s physical integrity and chemical stability during continuous column operation, making it highly suitable for fixed-bed adsorption systems. Table 5 presents a comparative overview of various adsorbents employed for the removal of MB.

**Table 5:** Comparative adsorption efficacy of some previously reported adsorbents for MB removal in a continuous system.

Adsorbents	q_eq_	Ref.
(PVA-GO) macroporous hydrogel bead	2.11	^90^
Alginate–water hyacinth bead	68.1	^91^
Activated Carbon-Infused Polyurethane	25.5	^41^
Polyurethane foam	4.00	^92^
Rice husk/CoFe_2_O_4_	16.26	^93^
Poly(Acrylonitrile-Co-Acrylic Acid) modified with thiourea	36.48	^94^
Calcium alginate-activated carbon	40.7	^95^
PVA/MCC–S–VO_2_ (PVA/MSV)	47.7 ± 1.8	This study

Error margins for literature values were included when reported in the cited references; otherwise, only mean values are presented.

#### Proposed mechanism for MB adsorption over PVA/MSV

**Fig. 15:**
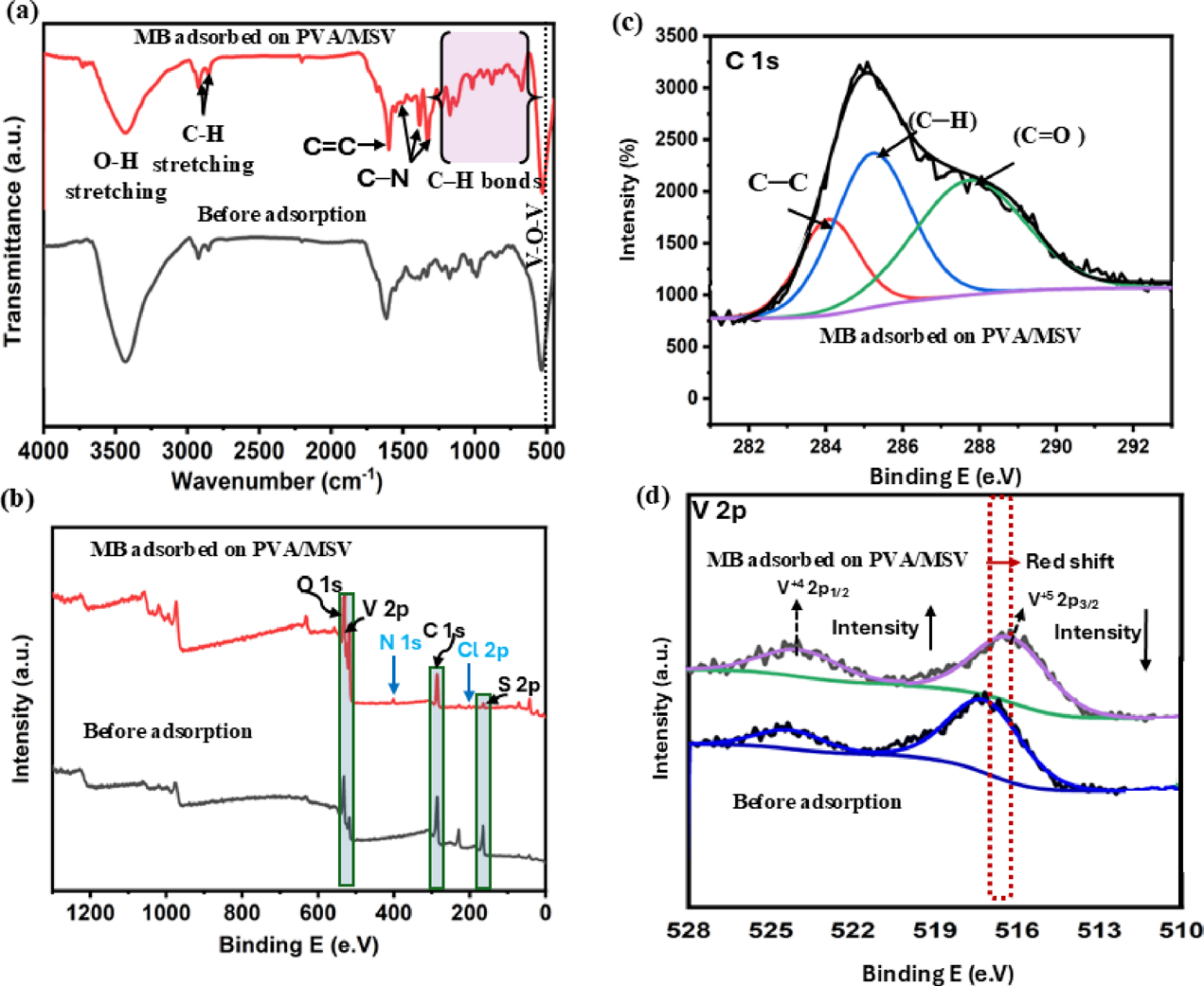
FT-IR (a) and XPS analysis (b─d) of PVA/MSV composite after methylene blue (MB) adsorption.

To improve our understanding of the sorption mechanism, the granules PVA/MSV were characterized using FTIR analysis (Fig. 15a) and XPS (Fig. 15b-d) following dye adsorption. The FTIR spectrum measurements revealed the existence of multiple significant functional groups on the surface of PVA/MSV. The broadband at around 3300–3600 cm^−1^ in PVA/MSV showed a noticeable decrease in intensity after MB adsorption, suggesting the potential participation of OH groups in the adsorption process. The peak associated with the aromatic ring vibration at 1600 cm^−1^ was highly pronounced. The prominent bands detected at 1490, 1395, and 1339 cm^−1^ were attributable to C–N stretching of the aromatic amine of the MB dye^94,95^. Multiple weak peaks ranging from 1253 to 669 cm^−1^ were attributed to the in-plane and out-of-plane bending vibrations of C–H bonds^96^. Notable changes in the intensity and position of characteristic absorption bands were observed, indicating potential interactions between the dye molecules and functional groups present in the composite. A minor red shift in the V–O–V stretching vibration band occurs, transitioning from 534 in pure PVA/MSV to 529 cm^−1^ in the composite after dye adsorption. The decrease in wavenumber signified a weakening or distortion of the V–O–V bonds, attributable to interactions between the VO_2_ surface and MB molecules. These interactions likely entail coordination between the lone pair electrons on MB, particularly from nitrogen or sulfur atoms, and the unoccupied d-orbitals of vanadium, as well as electrostatic interactions and hydrogen bonding.

**Fig. 16:**
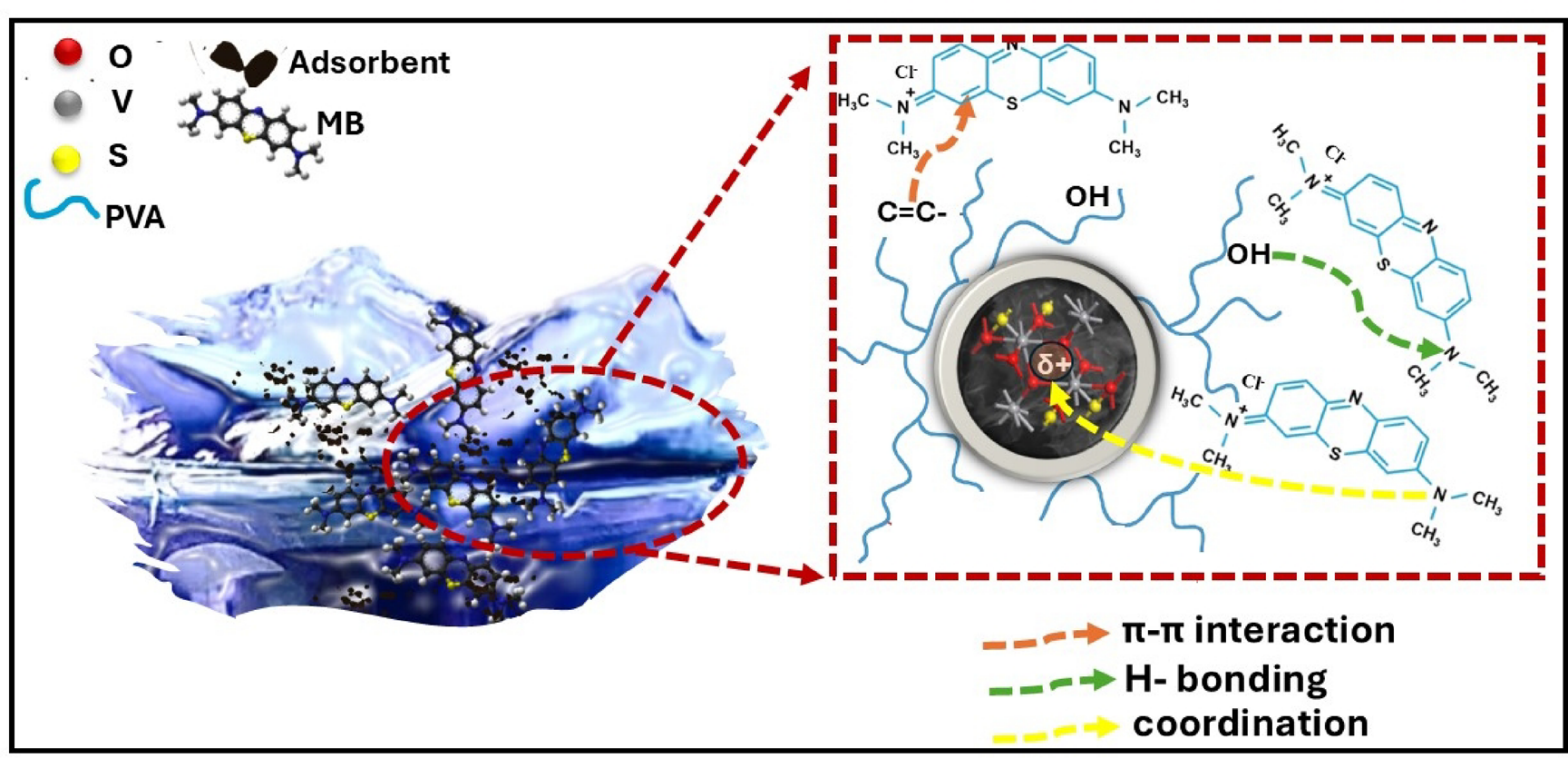
Proposed mechanism of methylene blue (MB) adsorption onto the PVA/MSV nanocomposite. The water image is an original photo taken by the authors for illustration.

The XPS spectrum of the PVA/MSV composite after MB adsorption showed the same main peaks as before adsorption, including carbon (C 1 s), oxygen (O 1 s), vanadium (V 2p), and sulfur (S 2p) as shown in Fig. 15b. The adsorption of MB onto the composite surface was confirmed by the detection of new peaks for nitrogen (N 1 s) and chlorine (Cl 2p), which are typical of the dye’s molecular structure. The C1s spectra of PVA/MSV (Fig. 5c) were deconvoluted into four peaks, with C–C at 284.55 eV and C–H at 285.28 eV. PVA thermally treated exhibits a third peak at 287.87 eV corresponding to the C = O bond and a fourth peak at 291.2 eV associated with the π-π* transition of aliphatic C = C bonds. However, the π–π* satellite peak at 291.2 eV was undetected after adsorption, suggesting that the π–π stacking interaction may have facilitated the adsorption of MB onto PVA/MSV, as shown in Fig. 15c. The V 2p region had reduced binding energies due to electron donation from MB’s amine groups to vanadium centers. A redox mechanism at the surface causes partial reduction of V^5+^ to V^4+^, resulting in a decrease in the V^5+^/V^4+^ ratio, which shows vanadium chemistry changes, demonstrating surface complexation or coordination after dye uptake. Importantly, the persistence of the monoclinic VO_2_ phase after calcination and repeated adsorption–regeneration cycles suggests that the carbonaceous PVA/MCC-derived matrix and sulfur play a protective role, suppressing excessive surface oxidation of VO_2_. This surface oxidation suppression mechanism ensures the stability of active vanadium sites, thereby enhancing recyclability and maintaining adsorption performance^97^. Complementary Raman spectroscopy (SI. Fig. S7) revealed a dominant band at ~ 1500–1600 cm^−1^, attributable to aromatic C = C stretching and C–N vibrations of MB ^99^. The persistence and slight shift of this band in the composite compared to free MB confirm π–π stacking and donor–acceptor interactions with the PVA/MSV matrix. Collectively, the FTIR, XPS, and Raman results provide converging evidence that MB adsorption proceeds via multi-step interactions involving hydrogen bonding, π–π stacking, and chemisorption through coordination with vanadium sites. As detailed in Fig. 16, the proposed mechanism supported the interaction pathways discussed.

## Conclusion

This study introduces an innovative melt intercalation technique for synthesizing PVA-encapsulated MCC/S–VO_2_ composite. The resulting granules exhibited a unique cross-linked network and diverse functional groups, contributing to outstanding methylene blue (MB) adsorption performance in fixed-bed systems. The kinetic behavior of PVA/MSV granules aligned closely with the BDST model (R^2^ > 0.9), indicating a surface-driven sorption mechanism. Optimized adsorption was achieved under conditions of increased bed height and reduced flow rate, particularly effective for treating wastewater containing low MB concentrations. The solvent-free melt intercalation method for producing the PVA/MSV composite offered a sustainable and highly efficient route for dye removal, well-suited for industrial applications. Additionally, the composite granules demonstrated remarkable reusability, easily retrievable from aqueous systems and viable for multiple use cycles—underscoring their economic and environmental advantages.

## Supplementary Information

Below is the link to the electronic supplementary material.

Supplementary Material 1

## Data Availability

This article incorporates all the data produced or examined during this study.

## References

[1] Alfuraydi, R. T., Al-Harby, N. F., Alminderej, F. M., Elmehbad, N. Y. & Mohamed, N. A. Poly (vinyl alcohol) hydrogels boosted with cross-linked Chitosan and silver nanoparticles for efficient adsorption of congo red and crystal Violet dyes. *Gels* **9**, 882 (2023). 37998972 10.3390/gels9110882 PMC10670830

[2] Elfiky, A. A. E. A., Mubarak, M. F., Keshawy, M., Sayed, I. E. T. E. & Moghny, T. A. Novel nanofiltration membrane modified by metal oxide nanocomposite for dyes removal from wastewater. *Environ. Dev. Sustain.* **26**, 19935–19957 (2024).

[3] Senguttuvan, S., Janaki, V., Senthilkumar, P. & Kamala-Kannan, S. Polypyrrole/zeolite composite–A nanoadsorbent for reactive dyes removal from synthetic solution. *Chemosphere* **287**, 132164 (2022). 34509762 10.1016/j.chemosphere.2021.132164

[4] Dos Santos, A. B., Cervantes, F. J. & Van Lier, J. B. Review paper on current technologies for decolourisation of textile wastewaters: perspectives for anaerobic biotechnology. *Bioresour Technol.* **98**, 2369–2385 (2007). 17204423 10.1016/j.biortech.2006.11.013

[5] El Gaayda, J. et al. Optimization of turbidity and dye removal from synthetic wastewater using response surface methodology: effectiveness of Moringa Oleifera seed powder as a green coagulant. *J. Environ. Chem. Eng.* **10**, 106988 (2022).

[6] Nassar, M. Y., NourEldien, M. S., Ibrahim, I. M. & Aly, H. M. A facile hydrothermal synthesis of S-VO_2_-cellulose nanocomposite for photocatalytic degradation of methylene blue dye. *Processes* **11**, 1322 (2023).

[7] Liu, H. et al. Biosynthesis based membrane filtration coupled with iron nanoparticles reduction process in removal of dyes. *Chem. Eng. J.* **387**, 124202 (2020).

[8] Zhang, K., Cui, C. & Wang, L. -j. Cellulose nanofiber-reinforced carboxymethyl chitosan/polyvinyl alcohol composite gels: physicochemical characterization and acid blue dye adsorption performance. *Int. J. Biol. Macromol.* **306**, 141695 (2025). 40044015 10.1016/j.ijbiomac.2025.141695

[9] Zhang, M., Xue, Y., Zhou, H., Xiang, A. & Deng, Y. Adsorption behaviors and mechanisms of Polyvinyl alcohol/xanthan gum/graphene oxide porous hydrogel for methylene blue and congo red. *Int. J. Biol. Macromol.* **308**, 142662 (2025). 40174821 10.1016/j.ijbiomac.2025.142662

[10] Zhao, S. et al. Efficient adsorption of anionic and cationic dyes by PVA/PAA/GO composite hydrogel with three-dimensional porous double network structure. *Mater. Chem. Phys.* **313**, 128716 (2024).

[11] Mashabi, R. A., Khan, Z. A. & Elwakeel, K. Z. Chitosan-or glycidyl methacrylate-based adsorbents for the removal of dyes from aqueous solutions: a review. *Mater. Adv.* **3**, 5645–5671 (2022).

[12] Santhi, T., Manonmani, S. & Ravi, S. Uptake of cationic dyes from aqueous solution by biosorption onto granular muntingia Calabura. *J. Chem.* **6**, 737–742 (2009).

[13] Graça, N. S., Ribeiro, A. M., Ferreira, A. & Rodrigues, A. E. *Application of Adsorption Processes for the Treatment of Diluted Industrial Effluents* (Springer, 2021).

[14] Gimenes, M. L., de Almeida Netob, A. F., Vieirab, M. G. & da Silvab, M. G. Continuous-flow copper adsorption in regenerable calcined clay columns. *Chem. Eng. J.* **32**, 2023–2028 (2013).

[15] Rashid, R., Shafiq, I., Akhter, P., Iqbal, M. J. & Hussain, M. A state-of-the-art review on wastewater treatment techniques: the effectiveness of adsorption method. *Environ. Sci. Pollut Res. Int.* **28**, 9050–9066 (2021). 33483933 10.1007/s11356-021-12395-x

[16] Uddin, M. T., Rukanuzzaman, M., Khan, M. M. R. & Islam, M. A. Adsorption of methylene blue from aqueous solution by jackfruit (Artocarpus heteropyllus) leaf powder: a fixed-bed column study. *J. Environ. Manage.* **90**, 3443–3450 (2009). 19541403 10.1016/j.jenvman.2009.05.030

[17] Hajiahmadi, Z., Moheb, A., Mohammadi, M., Marzban, N. & Scheufele, F. B. Surface and mass transfer kinetic and equilibrium modeling of Pb (II) ions adsorption on hydroxyapatite scaffold: batch and fixed-bed column studies. *Sep. Purif. Technol.* **343**, 127141 (2024).

[18] Rouf, S. & Nagapadma, M. Modeling of fixed bed column studies for adsorption of Azo dye on Chitosan impregnated with a cationic surfactant. *Int. J. Sci. Eng. Res.* **6**, 124–132 (2015).

[19] Long, Y. et al. Removal of Pb (Ⅱ) from aqueous solution by hydroxyapatite/carbon composite: Preparation and adsorption behavior. *Colloids Surf. A: Physicochem Eng. Asp*. **577**, 471–479 (2019).

[20] Valverde, A., Cabrera-Codony, A., Calvo-Schwarzwalder, M. & Myers, T. G. Investigating the impact of adsorbent particle size on column adsorption kinetics through a mathematical model. *Int. J. Heat. Mass. Transf.* **218**, 124724 (2024).

[21] Takaluoma, E. & Samarina, T. *in Alkali-Activated Materials in Environmental Technology Applications* 97–111 (Elsevier, 2022).

[22] Ragab, A. H., Mettwally, B. S., Mubarak, M. F., Al-Ghamdi, A. & Hemdan, M. Eco-friendly electrospinning of recycled nylon 6, 12 waste for high-performance nonwoven nanofibers in sustainable textile applications. *J. Inorg. Organomet. Polym. Mater.* **34**, 1491–1505 (2024).

[23] Gürses, A. & Güneş, K. Preparation of polyethylene clay composites via melt intercalation using hydrophobic and superhydrophobic organoclays and comparison of their textural, mechanical and thermal properties. *Polymers* **16**, 272 (2024). 38276681 10.3390/polym16020272 PMC10819245

[24] Nguyen Phuoc, H., Le, Q. T., Pham, T. C. T. & Le, T. T. Synthesis of glue-free NaA zeolite granules from natural Kaolin for the adsorption of Pb (II) ions in aqueous solution using a fixed-bed column study. *ACS Omega*. **6**, 21024–21032 (2021). 34423210 10.1021/acsomega.1c02658 PMC8375085

[25] Khademolhosseini, M. R., Mobasherpour, I. & Ghahremani, D. Lead absorption by nano-hydroxyapatite granules in a fixed-bed column. *Chem. Chem. Technol.* **12**, 372–378 (2018).

[26] Pansuk, C. & Vinitnantharat, S. Investigations on the Fixed-bed column performance of acid brown 75 adsorption by surface modified fly Ash granules. *Adv. Mater. Res.* **931**, 241–245 (2014).

[27] Wu, Y. et al. Optimized scalable synthesis and granulation of MIL-88B (Fe) for efficient arsenate removal. *J. Environ. Chem. Eng.* **10**, 108556 (2022).

[28] Kumar, J. & D’Souza, S. Preparation of PVA membrane for immobilization of GOD for glucose biosensor. *Talanta* **75**, 183–188 (2008). 18371866 10.1016/j.talanta.2007.10.048

[29] Ermakova, M. A. & Ermakov, D. Y. High-loaded nickel–silica catalysts for hydrogenation, prepared by sol–gel: route: structure and catalytic behavior. *Appl. Catal. A-Gen*. **245**, 277–288 (2003).

[30] Jia, F., Zhang, L., Shang, X. & Yang, Y. Non-Aqueous Sol–Gel approach towards the controllable synthesis of nickel Nanospheres, Nanowires, and nanoflowers. *Adv. Mater.* **20**, 1050–1054 (2008).

[31] Morishita, T., Soneda, Y., Tsumura, T. & Inagaki, M. Preparation of porous carbons from thermoplastic precursors and their performance for electric double layer capacitors. *Carbon* **44**, 2360–2367 (2006).

[32] Geng, J., Qin, J. & He, J. Preparation of intercalated organic montmorillonite DOPO-MMT by melting method and its effect on flame retardancy to epoxy resin. *Polymers* **13**, 3496 (2021). 34685257 10.3390/polym13203496 PMC8539982

[33] Couți, N. et al. Polyvinyl Alcohol, a versatile excipient for pharmaceutical 3D printing. *Polymers* **16**, 517 (2024). 38399895 10.3390/polym16040517 PMC10893462

[34] Naduparambath, S. et al. Development of green composites of Poly (vinyl alcohol) reinforced with microcrystalline cellulose derived from Sago seed shells. *Polym. Compos.* **39**, 3033–3039 (2018).

[35] Das, K. et al. A study of the mechanical, thermal and morphological properties of microcrystalline cellulose particles prepared from cotton slivers using different acid concentrations. *Cellulose* **16**, 783–793 (2009).

[36] Valmalette, J. C. & Gavarri, J. R. High efficiency thermochromic VO_2_ (R) resulting from the irreversible transformation of VO_2_ (B). *Mater. Sci. Eng. B*. **54**, 168–173 (1998).

[37] Patel, H. Fixed-bed column adsorption study: a comprehensive review. *Appl. Water Sci.* **9**, 45 (2019).

[38] Chen, S. et al. Adsorption of hexavalent chromium from aqueous solution by modified corn stalk: a fixed-bed column study. *Bioresour Technol.* **113**, 114–120 (2012). 22189077 10.1016/j.biortech.2011.11.110

[39] Srikanth, K., King, P. & Pujari, M. Breakthrough studies of the adsorption of lead from synthetic solution using Liagora viscida in a fixed bed column. *Environ. Prog Sustain. Energy*. **40**, e13628 (2021).

[40] Acheampong, M. A., Pakshirajan, K., Annachhatre, A. P. & Lens, P. N. Removal of Cu (II) by biosorption onto coconut shell in fixed-bed column systems. *J. Ind. Eng. Chem.* **19**, 841–848 (2013).

[41] Estrada, R. J. R. et al. Sequestration of methylene blue dye in a Fixed-Bed column using activated Carbon-Infused polyurethane composite adsorbent derived from coconut oil. *Sustainability* **16**, 10757 (2024).

[42] Halim, A. A., Aziz, H. A., Johari, M. A. M., Ariffin, K. S. & Adlan, M. N. Ammoniacal nitrogen and COD removal from semi-aerobic landfill leachate using a composite adsorbent: fixed bed column adsorption performance. *J. Hazard. Mater.* **175**, 960–964 (2010). 19945216 10.1016/j.jhazmat.2009.10.103

[43] Cruz-Olivares, J. et al. Modeling of lead (II) biosorption by residue of allspice in a fixed-bed column. *Chem. Eng. J.* **228**, 21–27 (2013).

[44] Bohart, G. & Adams, E. Some aspects of the behavior of charcoal with respect to Chlorine. *J. Am. Chem. Soc.* **42**, 523–544 (1920).

[45] Sadaf, S. & Bhatti, H. N. Evaluation of peanut husk as a novel, low cost biosorbent for the removal of Indosol orange RSN dye from aqueous solutions: batch and fixed bed studies. *Clean. Technol. Environ. Policy*. **16**, 527–544 (2014).

[46] Thomas, H. C. Heterogeneous ion exchange in a flowing system. *J. Am. Chem. Soc.* **66**, 1664–1666 (1944).

[47] Yoon, Y. H. & Nelson, J. H. Application of gas adsorption kinetics I. A theoretical model for respirator cartridge service life. *Am. Ind. Hyg. Assoc. J.* **45**, 509–516 (1984). 6475758 10.1080/15298668491400197

[48] Ahmad, A. & Hameed, B. Fixed-bed adsorption of reactive Azo dye onto granular activated carbon prepared from waste. *J. Hazard. Mater.* **175**, 298–303 (2010). 19883979 10.1016/j.jhazmat.2009.10.003

[49] Aziz, S. B. Modifying Poly (vinyl alcohol)(PVA) from insulator to small-bandgap Polymer: A novel approach for organic solar cells and optoelectronic devices. *J. Electron. Mater.* **45**, 736–745 (2016).

[50] Abdullah, O. G., Aziz, S. B. & Rasheed, M. A. Structural and optical characterization of PVA: KMnO4 based solid polymer electrolyte. *Results Phys.* **6**, 1103–1108 (2016).

[51] Kesavan, K., Rajendran, S. & Mathew, C. M. Studies on Poly (vinyl pyrrolidone) based solid Polymer blend electrolytes complexed with various lithium salts. *Polym. Sci. Ser. B*. **56**, 520–529 (2014).

[52] Abdelfattah, E. et al. Enhancement of the structure, thermal, linear/nonlinear optical properties, and antibacterial activity of Poly (vinyl alcohol)/chitosan/ZnO nanocomposites for eco-friendly applications. *Polymers* **15**, 4282 (2023). 37959962 10.3390/polym15214282 PMC10648650

[53] Jenkins, R. & Snyder, R. L. *Introduction to X-ray powder diffractometry* Vol. 138 (Wiley Online Library, 1996).

[54] Qian, X. et al. Polymer–inorganic nanocomposites prepared by hydrothermal method: Preparation and characterization of PVA–transition-metal sulfides. *J. Appl. Polym. Sci.* **82**, 2744–2749 (2001).

[55] Yang, H., Xu, S., Jiang, L. & Dan, Y. Thermal decomposition behavior of Poly (vinyl alcohol) with different hydroxyl content. *J. Macromol. Sci. Part. B*. **51**, 464–480 (2012).

[56] Mubarak, M. F., Khedr, G. E. & El Sharkawy, H. M. Environmentally-friendly calcite scale mitigation: encapsulation of CDs@ MS composite within membranes framework for nanofiltration. *J. Alloys Compd.* **999**, 175061 (2024).

[57] Ji, H. et al. Infrared thermochromic properties of monoclinic VO_2_ nanopowders using a malic acid-assisted hydrothermal method for adaptive camouflage. *RSC Adv.* **7**, 5189–5194 (2017).

[58] Frost, R. L., Locke, A. J., Hales, M. C. & Martens, W. N. Thermal stability of synthetic aurichalcite implications for making mixed metal oxides for use as catalysts. *J. Therm. Anal. Calorim.* **94**, 203–208 (2008).

[59] Kim, H., Youn, J. R. & Song, Y. S. Eco-friendly flame retardant nanocrystalline cellulose prepared via silylation. *Nanotechnology* **29**, 1–21 (2018). 10.1088/1361-6528/aadc87 30136647

[60] Cobos, M. & Fernández, M. Graphene based Poly (Vinyl Alcohol) nanocomposites prepared by in situ green reduction of graphene oxide by ascorbic acid: influence of graphene content and glycerol plasticizer on properties. *Nanomaterials* **8**, 1013 (2018). 30563225 10.3390/nano8121013 PMC6316035

[61] Tămăşan, M., Radu, T. & Simon, V. Spectroscopic characterisation and in vitro behaviour of kaolinite Polyvinyl alcohol nanocomposite. *Appl. Clay Sci.* **72**, 147–154 (2013).

[62] Zhang, C., Sunarso, J. & Liu, S. Designing CO_2_-resistant oxygen-selective mixed ionic–electronic conducting membranes: guidelines, recent advances, and forward directions. *Chem. Soc. Rev.* **46**, 2941–3005 (2017). 28436504 10.1039/c6cs00841k

[63] Smart, R. S. C., Skinner, W. M. & Gerson, A. R. XPS of sulphide mineral surfaces: metal-deficient, polysulphides, defects and elemental sulphur. *Surf. Interface Anal.* **28**, 101–105 (1999).

[64] Silversmit, G., Depla, D., Poelman, H., Marin, G. B. & De Gryse, R. Determination of the V2p XPS binding energies for different vanadium oxidation States (V^5+^ to V^0+^). *J. Electron. Spectrosc.* **135**, 167–175 (2004).

[65] Wang, J. et al. Lightweight, interconnected VO_2_ nanoflowers hydrothermally grown on 3D graphene networks for wide-voltage-window supercapacitors. *RSC Adv.* **7**, 35558–35564 (2017).

[66] Zou, Z., Li, N. & Li, D. Corrosion protection properties of vanadium films formed on zinc surfaces. *Rare Met.* **30**, 146–149 (2011).

[67] Bondarenka, V., Grebinskij, S., Kačiulis, S., Mattogno, G. & Mickevičius, S. Surface chemical composition of MV_10_Mo_2_O_31_· nH_2_O (M = Na_2_, K_2_, Ca, Sr, Cu) xerogels. *J. Electron. Spectrosc. Relat. Phenom.* **107**, 253–259 (2000).

[68] Riyadh, S. M., Khalil, K. D. & Bashal, A. H. Structural properties and catalytic activity of binary Poly (vinyl alcohol)/Al2O3 nanocomposite film for synthesis of thiazoles. *Catalysts* **10**, 100 (2020).

[69] Wu, S. et al. Influence of surface roughness on cetyltrimethylammonium bromide adsorption from aqueous solution. *Langmuir* **27**, 6091–6098 (2011). 21488630 10.1021/la200751m

[70] Song, S., Huang, Q. & Zhu, W. Hydrothermal route to VO_2_ (B) nanorods: controlled synthesis and characterization. *J. Nanoparticle Res.* **19**, 1–8 (2017).

[71] Crittenden, J. C., Berrigan, J. K. & Hand, D. W. Design of rapid small-scale adsorption tests for a constant diffusivity. *J. Water Pollut Control Fed.* **58**, 312–319 (1986).

[72] Schumann, P. et al. Pilot-scale removal of persistent and mobile organic substances in granular activated carbon filters and experimental predictability at lab-scale. *Sci. Total Environ.* **884**, 163738 (2023). 37116805 10.1016/j.scitotenv.2023.163738

[73] Zhuang, Y., Yu, F., Chen, J. & Ma, J. Batch and column adsorption of methylene blue by graphene/alginate nanocomposite: comparison of single-network and double-network hydrogels. *J. Environ. Chem. Eng.* **4**, 147–156 (2016).

[74] Kólová, A. & Stejskalová, L. A review Article of rapid Small-Scale column tests. *VTEI* **64**, 41–45 (2022).

[75] Chen, N. et al. Investigations on the batch and fixed-bed column performance of fluoride adsorption by Kanuma mud. *Desalination* **268**, 76–82 (2011).

[76] Baral, S. et al. Removal of cr (VI) by thermally activated weed Salvinia cucullata in a fixed-bed column. *J. Hazard. Mater.* **161**, 1427–1435 (2009). 18571842 10.1016/j.jhazmat.2008.04.127

[77] Cooney, D. O. External film and particle phase control of adsorber breakthrough behavior. *AIChE j.* **36**, 1430–1432 (1990).

[78] Ko, D. C., Porter, J. F. & McKay, G. Optimised correlations for the fixed-bed adsorption of metal ions on bone Char. *Chem. Eng. Sci.* **55**, 5819–5829 (2000).

[79] Vijayaraghavan, K., Jegan, J., Palanivelu, K. & Velan, M. Removal of nickel (II) ions from aqueous solution using crab shell particles in a packed bed up-flow column. *J. Hazard. Mater.* **113**, 223–230 (2004). 15363535 10.1016/j.jhazmat.2004.06.014

[80] Han, R. et al. Characterization and properties of iron oxide-coated zeolite as adsorbent for removal of copper (II) from solution in fixed bed column. *Chem. Eng. J.* **149**, 123–131 (2009).

[81] Boucherdoud, A., Kherroub, D., Bestani, B., Benderdouche, N. & Douinat, O. Fixed-bed adsorption dynamics of methylene blue from aqueous solution using alginate-activated carbon composites adsorbents. *Alger. J. Environ. Sci. Technol. Mon*. **8**, 2329–2337 (2022).

[82] Dotto, G. et al. Fixed bed adsorption of methylene blue by ultrasonic surface modified Chitin supported on sand. *Chem. Eng. Res. Des.* **100**, 302–310 (2015). 10.1016/j.jcis.2015.01.046 25660713

[83] Al-Janabi, N. et al. Velocity variation effect in fixed bed columns: A case study of CO_2_ capture using porous solid adsorbents. *AIChE J.* **64**, 2189–2197 (2018).

[84] Naboulsi, A. et al. Fixed-bed adsorption of pesticide agricultural waste using cross-linked adsorptive hydrogel composite beads. *Environ. Sci. Pollut Res.* **31**, 32320–32338 (2024). 10.1007/s11356-024-33388-6 38653892

[85] Elkholy, A. S., Yahia, M. S., Elnwawy, M. A., Gomaa, H. A. & Elzaref, A. S. Synthesis of activated carbon composited with Egyptian black sand for enhanced adsorption performance toward methylene blue dye. *Sci. Rep.* **13**, 4209 (2023). 36918583 10.1038/s41598-023-28556-6 PMC10015066

[86] Fangwen, L. et al. Adsorption and desorption mechanisms of methylene blue removal with iron-oxide coated porous ceramic filter. *J. Water Resour. Prot.* **1**, 35–40 (2009).

[87] Jayalakshmi, R. & Jeyanthi, J. Simultaneous removal of binary dye from textile effluent using Cobalt ferrite-alginate nanocomposite: performance and mechanism. *Microchem J.* **145**, 791–800 (2019).

[88] Chen, D., Zhou, J., Wang, H. & Yang, K. Batch and fixed-bed column study for p-nitrophenol, methylene blue, and U (VI) removal by Polyvinyl alcohol–graphene oxide macroporous hydrogel bead. *Water Sci. Technol.* **77**, 91–100 (2018). 29339607 10.2166/wst.2017.524

[89] Mahamadi, C. & Mawere, E. Continuous flow biosorptive removal of methylene blue and crystal Violet dyes using alginate–water hyacinth beads. *Cogent Environ. Sci.* **5**, 1594513 (2019).

[90] Baldez, E. E., Robaina, N. F. & Cassella, R. J. Employment of polyurethane foam for the adsorption of methylene blue in aqueous medium. *J. Hazard. Mater.* **159**, 580–586 (2008). 18395335 10.1016/j.jhazmat.2008.02.055

[91] Yan, K. K. et al. Fixed-bed adsorption of methylene blue by rice husk Ash and rice husk/CoFe_2_O_4_ nanocomposite. *desalin. Water Treat.* **57**, 12793–12803 (2016).

[92] Adeyi, A. A. et al. Simultaneous adsorption of malachite green and methylene blue dyes in a fixed-bed column using Poly (acrylonitrile-co-acrylic acid) modified with thiourea. *Molecules* **25**, 2650 (2020). 32517324 10.3390/molecules25112650 PMC7321146

[93] Boucherdouda, A., Bestani, B., Benderdouche, N. & Duclaux, L. The use of calcium alginate-activated carbon composite material in fixed-bed columns for methylene blue removal from wastewater. *Desalin. Water Treat.* **154**, 356–368 (2019).

[94] Balu, P., Asharani, I. & Thirumalai, D. Catalytic degradation of hazardous textile dyes by iron oxide nanoparticles prepared from Raphanus sativus leaves’ extract: a greener approach. *J. Mater. Sci. Mater. Electron.* **31**, 10669–10676 (2020).

[95] Xiong, L. et al. Adsorption behavior of methylene blue onto titanate nanotubes. *J. Chem. Eng.* **156**, 313–320 (2010).

[96] Yan, Y. et al. Adsorption of methylene blue dye onto carbon nanotubes: a route to an electrochemically functional nanostructure and its layer-by-layer assembled nanocomposite. *Chem. Mater.* **17**, 3457–3463 (2005).

[97] Zhu, H. et al. The regulatory role of dissolved oxygen in N-doped biochar-driven nonradical oxidation. *Chem. Eng. J.* **520**, 165915 (2025).

[98] Tu, K. & Chung, C. Enhancement of surface Raman spectroscopy performance by silver nanoparticles on resin nanorods arrays from anodic aluminum oxide template. *J. Electrochem. Soc.* **164**, B3081 (2017).

