# Supplementary material for: Fabrication of a PVA-encapsulated MCC/S–VO2 composite via melt intercalation for efficient fixed-bed adsorption of methylene blue
Source: Sci Rep. 2025 Oct 29;15:37744. doi: 10.1038/s41598-025-22645-4 (PMC12572330; doi:10.1038/s41598-025-22645-4)
Supplement: Supplementary file 1 — Supplementary Material 1 [file 41598_2025_22645_MOESM1_ESM.docx]

Supplementary Information (SI)

**Fabrication of a PVA-encapsulated MCC/S–VO_2_ composite via melt intercalation for efficient fixed-bed adsorption of methylene blue**

Mona S. NourEldien^1*^, Hisham M. Aly^1^

^1^Department of Chemistry, Faculty of Science, Benha University, Benha 13518, Egypt

*Corresponding author.

E-mail address: [mona.noureldin@fsc.bu.edu.eg](mailto:mona.noureldin@fsc.bu.edu.eg)


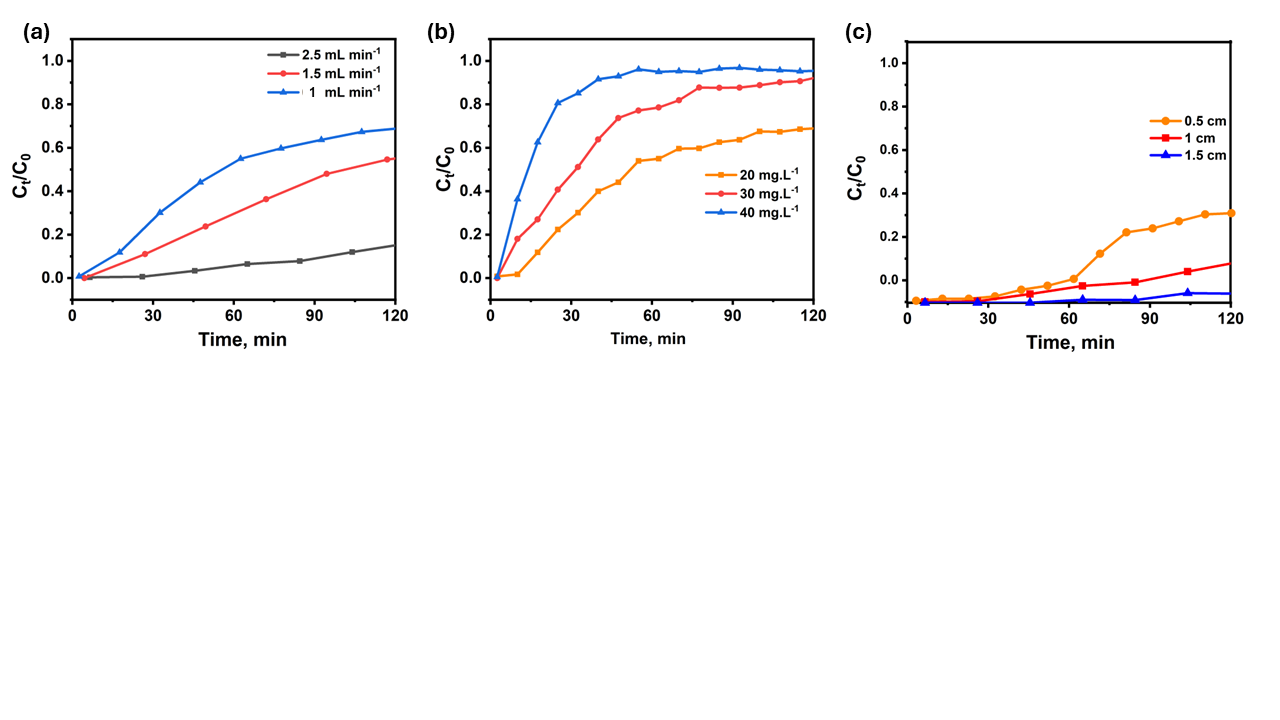


**Fig. S1**. Screening breakthrough curves for methylene blue (MB) adsorption in PVA–MSV fixed-bed columns. Short runs (~2 h) were performed by varying (a) flow rate (1.0–2.5 mL min^-1^, H = 1.0 cm), (b) influent MB concentration (20–40 mg L^-1^), and (c) bed height (0.5–1.5 cm, Q = 1.5 mL min^-1^).

**Table S1.** Calculated empty bed contact times (EBCT) and observed breakthrough times for screening experiments under different bed heights and flow rates (column i.d. = 1.0 cm).

| **Bed height (cm)** | **Bed volume (mL)** | **EBCT (min)** | **t_b_ (min)** |
| --- | --- | --- | --- |
| **Flow rate= 1.5 mL min^-1^, dye conc.= 20 mg L^-1^** |  |  |  |
| 0.5 | 0.393 | 0.262 | 62 |
| 1 | 0.785 | 0.524 | Not reached |
| 1.5 | 1.178 | 0.785 | Not reached |
| **Flow rate (mL min^-1^)** | **Bed volume (mL)** | **EBCT (min)** | **t_b_ (min)** |
| **Bed height = 1 cm, dye conc.= 20 mg L^-1^** |  |  |  |
| 1 | 0.785 | 0.785 | 17 |
| 1.5 | 0.785 | 0.524 | 25 |
| 2.5 | 0.785 | 0.314 | 106 |

**Table S2**: Summary of the fitting parameters, including binding energies, FWHM values, and relative areas.

| **Component** | **BE (eV)** | **FWHM (eV)** | **Relative Area (%)** | **Assignment** |
| --- | --- | --- | --- | --- |
| O1 | 529.73 ± 0.1 | 1.16 | 3.9 ± 0.5 | Lattice oxygen (O^2-^ in VO_2_) |
| O2 | 531.18 ± 0.1 | 2.31 | 45.2 ± 2.0 | O in the crystalline structure |
| O3 | 532.91 ± 0.1 | 2.53 | 25.0 ± 1.5 | Carbonyl (from PVA/cellulose) |
| O4 | 534.47 ± 0.2 | 3.37 | 25.9 ± 1.5 | O in the adsorbed water |
| V 2p_3/2_ (V^4+^) | 516.4 ± 0.2 | 2.95 ± 0.2 | 43.7 ± 2.0 | V^4+^ in VO_2_ |
| V 2p_3/2_  (V^5+^) | 517.1 ± 0.2 | 2.81 ± 0.2 | 48.2 ± 2.0 | V^5+^ species (surface oxidation) |
| V 2p_1/2_  (V^4+^) | 523.4 ± 0.2 | 2.95 ± 0.2 | 9.0 ± 1.0 | V^4+^ in VO_2_ |
| V 2p_1/2_  (V^5+^) | 524.4 ± 0.2 | 2.76 ± 0.2 | 12.1 ± 1.0 | V^5+^ species (surface oxidation) |
| S 2p_3/2_ | 164.5 ± 0.1 | 2.42 ± 0.2 | 66.4 ± 2.0 | Sulfide (S^2-^) in S–VO_2_ framework |
| S 2p_1/2_ | 167.5 ± 0.1 | 3.37 ± 0.2 | 33.6 ± 2.0 | Oxidized sulfur species (SO_x_, surface oxidation) |
| C–C | 284.6 ± 0.1 | 1.85 ± 0.1 | 18.3 ± 1.0 | Hydrocarbon backbone (PVA, MCC) |
| C–H | 285.3 ± 0.1 | 2.35 ± 0.2 | 38.0 ± 2.0 | Hydrocarbon backbone (PVA, MCC) |
| C=O | 287.9 ± 0.1 | 3.37 ± 0.2 | 38.4 ± 2.0 | Carbonyl, oxidation species |
| C=C | 291.2 ± 0.1 | 1.05 ± 0.1 | 0.8 ± 0.1 | π-π* transition of aliphatic C=C |


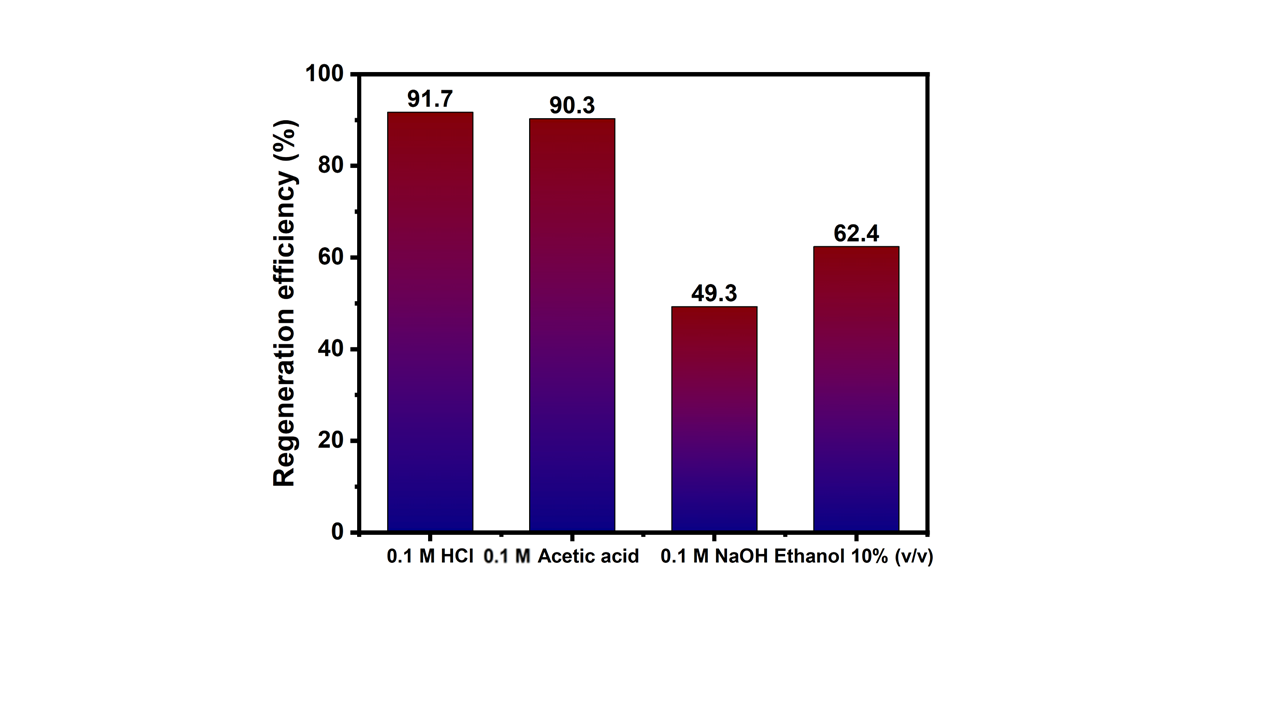


**Fig. S2** Preliminary regeneration screening of PVA/MSV adsorbent using different eluents (0.1 M HCl, 0.1 M acetic acid, ethanol, and 0.1 M NaOH). (dye conc. = 20 mg L^-1^, flow rate = 1 mL min^-1^, bed height = 0.5 cm).


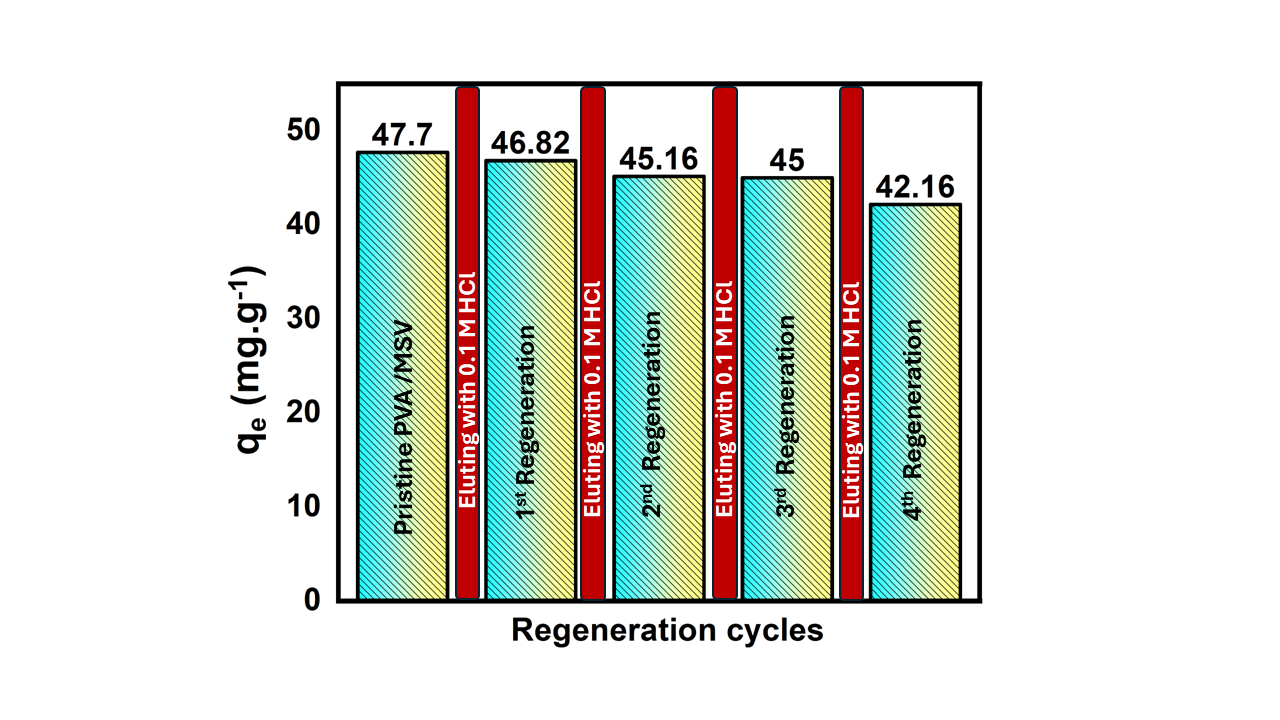


**Fig. S3** Regeneration experiments of PVA/MSV (for the effective removal of methylene blue (MB): (dye conc. = 20 mg L^-1^, flow rate = 1 mL min^-1^, bed height = 0.5 cm). optimized regeneration conditions ((i) extended static soaking (50 min) and (ii) reduced-flow circulation (1.0 mL min^-1^) combined with 10 min ultrasonic assist).


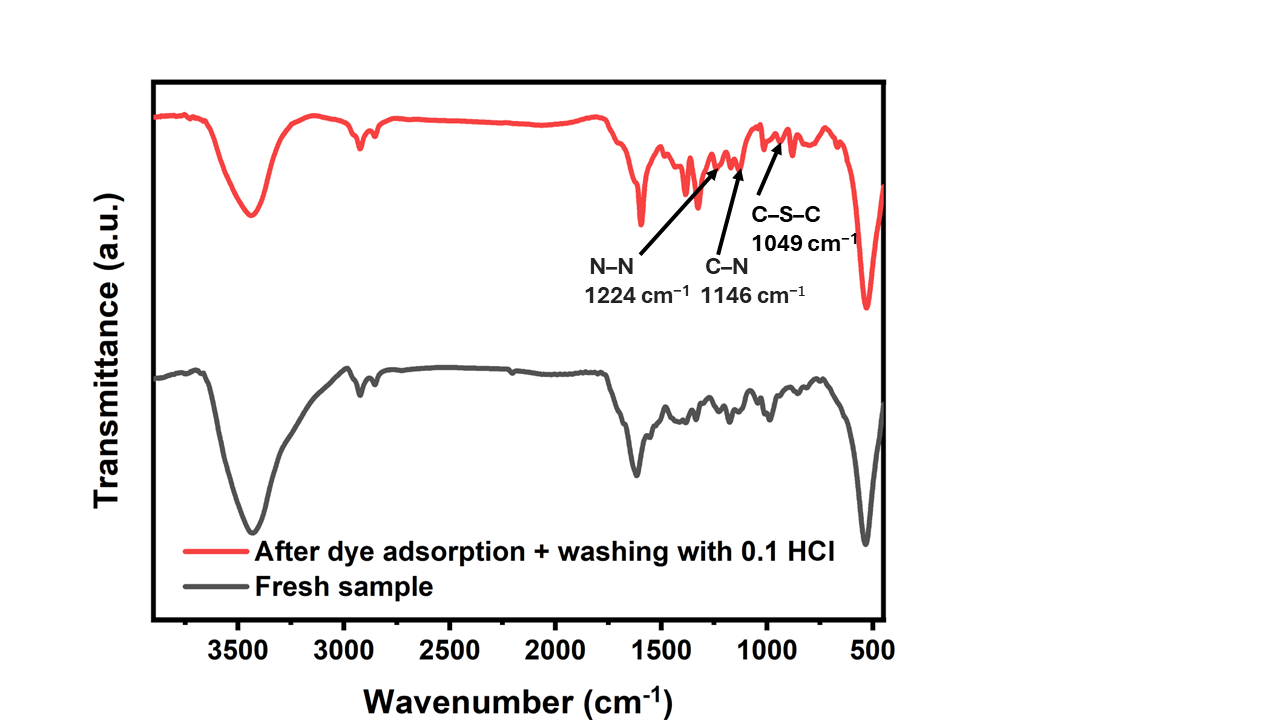


**Fig. S4.** FTIR spectra of the regenerated PVA/MSV.


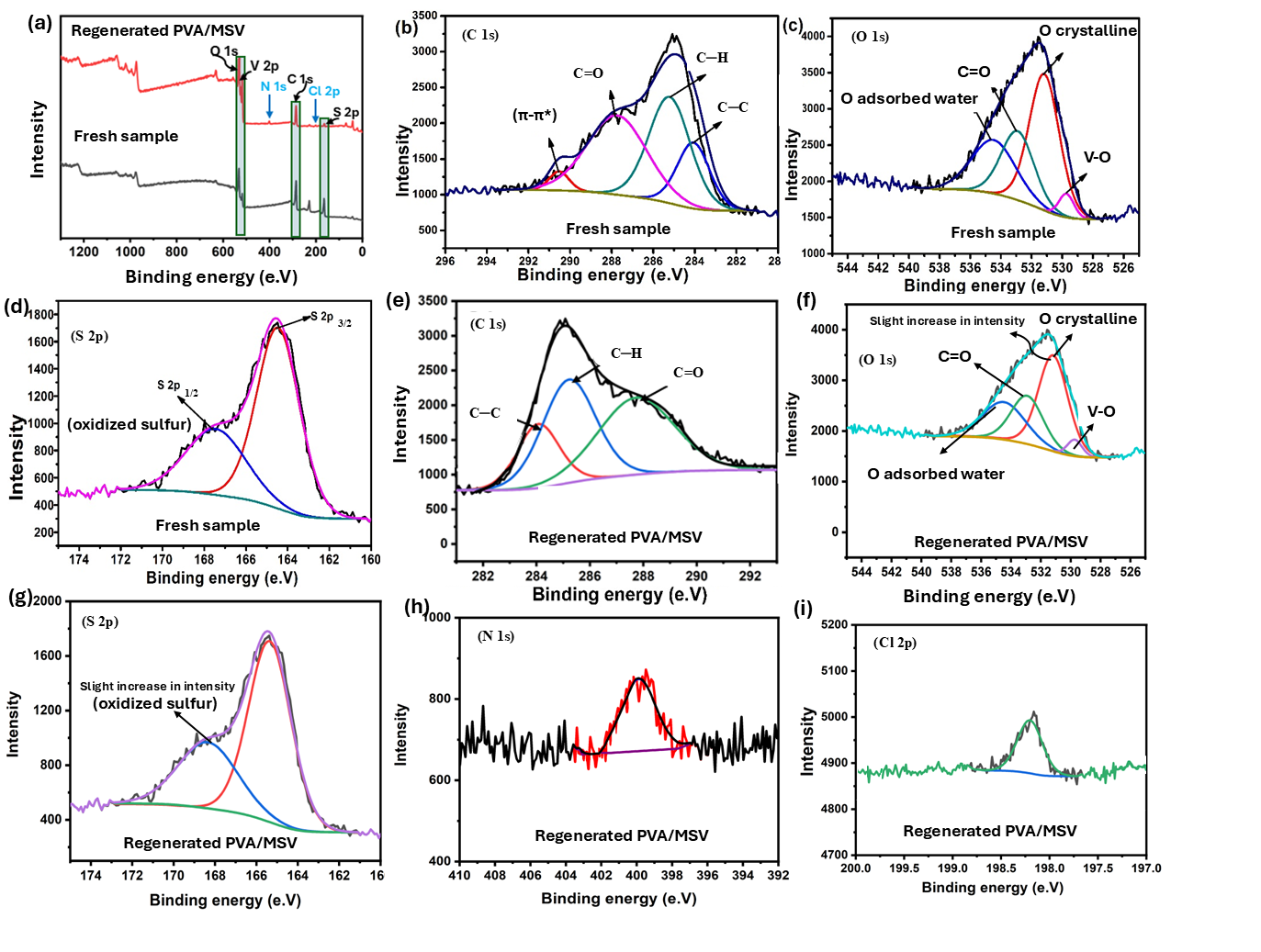


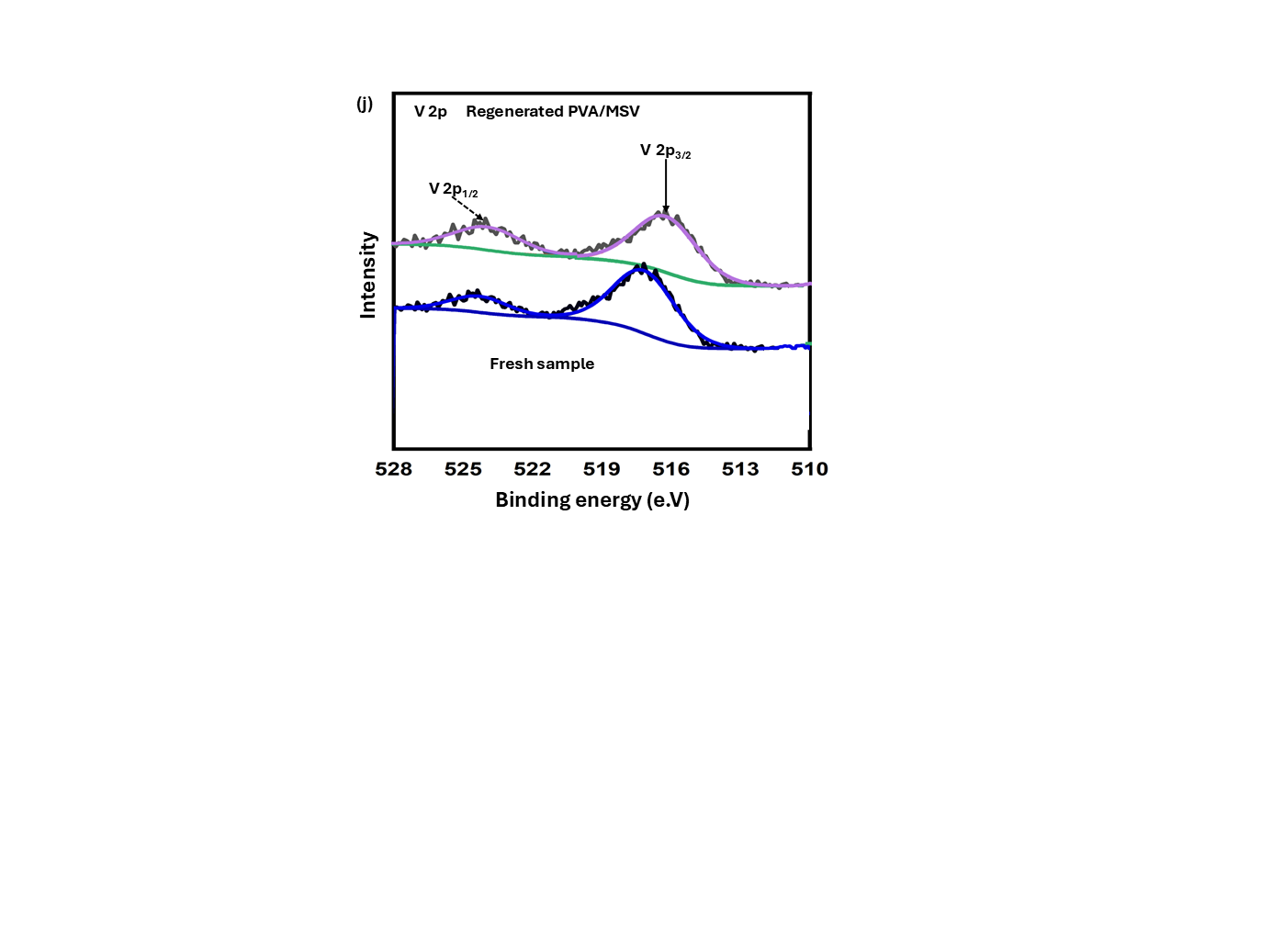


**Fig. S5** XPS spectra of regenerated PVA/MSV composite. Full spectrum (a), C 1s (b&e), O 1s (c&f), S 2p (d&g), N 1s (h), Cl 2p (i), and V 2p (j).


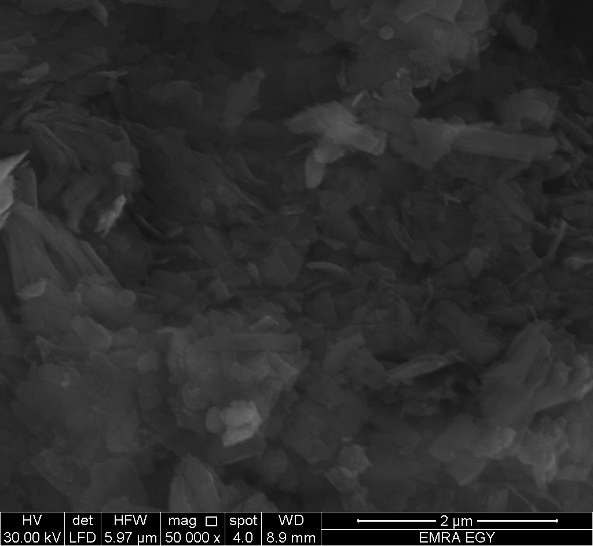


**Fig. S6** FE-SEM for PVA/MSV composite after 4 adsorption cycles.


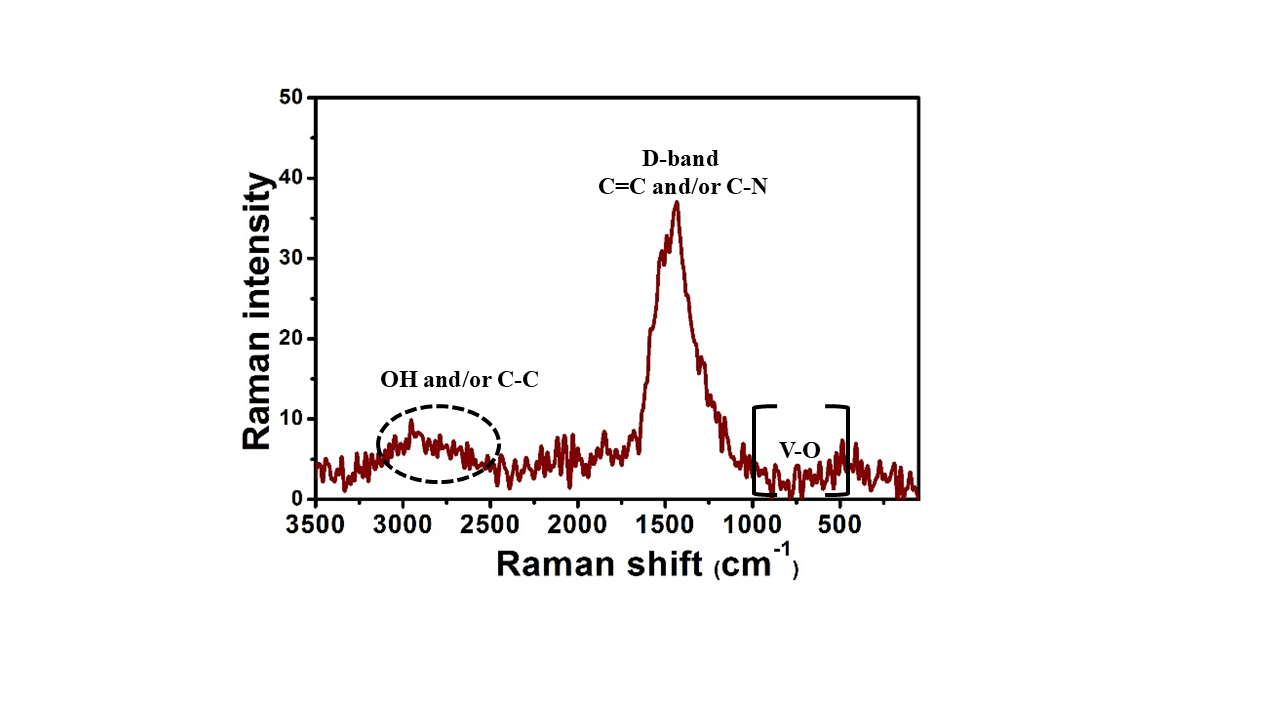


**Fig. S7** Raman spectrum of PVA/MSV after MB adsorption.
